# Chimeric Human Papillomavirus-16 Virus-like Particles Presenting HIV-1 P18I10 Peptide: Expression, Purification, Bio-Physical Properties and Immunogenicity in BALB/c Mice

**DOI:** 10.3390/ijms24098060

**Published:** 2023-04-29

**Authors:** Chun-Wei Chen, Narcís Saubi, Joan Joseph-Munné

**Affiliations:** 1 Department of Biomedical Sciences, University of Barcelona, 08036 Barcelona, Spain; cwchen0927@gmail.com; 2 Vall d’Hebron Research Institute (VHIR), 08035 Barcelona, Spain; 3 Department of Microbiology, Hospital Universitari Vall d’Hebron, 08035 Barcelona, Spain; 4 Respiratory Viruses Unit, Virology Section, Microbiology Department, Hospital Universitari Vall d’Hebron, 08035 Barcelona, Spain

**Keywords:** HIV-1, HPV16, virus-like particle, vaccines, BEVS/IC system, mammalian 293F cell expression system, sucrose cushion, cesium chloride gradients, chromatography, immunogenicity

## Abstract

Human papillomavirus (HPV) vaccines based on HPV L1 virus-like particles (VLPs) are already licensed but not accessible worldwide. About 38.0 million people were living with HIV in 2020 and there is no HIV vaccine yet. Therefore, safe, effective, and affordable vaccines against both viruses are an urgent need. In this study, the HIV-1 P18I10 CTL peptide from the V3 loop of HIV-1 gp120 glycoprotein was inserted into the HPV16 L1 protein to construct chimeric HPV:HIV (L1:P18I10) VLPs. Instead of the traditional baculovirus expression vector/insect cell (BEVS/IC) system, we established an alternative mammalian 293F cell-based expression system using cost-effective polyethylenimine-mediated transfection for L1:P18I10 protein production. Compared with conventional ultracentrifugation, we optimized a novel chromatographic purification method which could significantly increase L1:P18I10 VLP recovery (~56%). Chimeric L1:P18I10 VLPs purified from both methods were capable of self-assembling to integral particles and shared similar biophysical and morphological properties. After BALB/c mice immunization with 293F cell-derived and chromatography-purified L1:P18I10 VLPs, almost the same titer of anti-L1 IgG (*p* = 0.6409) was observed as Gardasil anti-HPV vaccine-immunized mice. Significant titers of anti-P18I10 binding antibodies (*p* < 0.01%) and P18I10-specific IFN-γ secreting splenocytes (*p* = 0.0002) were detected in L1:P18I10 VLP-immunized mice in comparison with licensed Gardasil-9 HPV vaccine. Furthermore, we demonstrated that insertion of HIV-1 P18I10 peptide into HPV16 L1 capsid protein did not affect the induction in anti-L1 antibodies. All in all, we expected that the mammalian cell expression system and chromatographic purification methods could be time-saving, cost-effective, scalable platforms to engineer bivalent VLP-based vaccines against HPV and HIV-1

## 1. Introduction

Human immunodeficiency virus-1 (HIV-1), which causes acquired immunodeficiency syndrome (AIDS), was discovered in the early 1980s, and since then, it became a global pandemic [1]. An effective HIV vaccine should elicit broadly neutralizing antibodies, innate and adaptive long-lasting mucosal immunity [2], including potent stimulation of CD4+ [3] and cytotoxic CD8+ T lymphocytes to prevent HIV-1 infection [4]. In the most successful case, the RV144 Thailand trial revealed a modest efficacy of 31.2% against HIV-1 acquisition [5]. The majority of previous HIV-1 vaccine candidates that underwent clinical trials were mainly based on subunit protein, DNA, and recombinant viral vector vaccine models [6,7]. However, a licensed virus-like particle (VLP)-based HIV-1 vaccine until now is still unavailable.

Over a hundred types of human papilloma virus (HPV) were already known and the HPV genotypes 16 and 18 are defined to be the cause for about 70% of women cervical cancer all over the world [8]. HPV L1 VLPs, classified as a type of subunit vaccine, can predominantly elicit L1-specific antibody responses and cellular responses to HPV virion [9,10,11]. Recently, three prophylactic HPV vaccines based on VLPs of HPV L1 capsid protein were commercialized on the market. The Cervarix (GSK) was produced by using the baculovirus expression vector/insect cell (BEVS/IC) system, while the Gardasil (Merck), Gardasil-9 (Merck) were produced by the yeast (Saccharomyces cerevisiae) expression system [12]. However, these three are really expensive for most women in developing countries. Thus, additional efforts, such as the development of safe and affordable HPV and HIV vaccines, must be carried out to achieve proper prevention and control of new HPV and HIV infections.

In our prior review papers, titled design concepts of VLP-based HIV-1 and HPV vaccines, we mentioned that papillomavirus VLPs, defined as non-enveloped VLPs, could play a functional role as delivery vehicles to present HIV-1 neutralizing antibody or CTL epitopes [13,14]. This hypothesis was demonstrated in many chimeric bovine papillomavirus (BPV) L1 VLP presenting 2F5 epitope or MPER region of HIV-1 envelope (Env) gp41 and also P18I10 CTL epitope from third variable domain (V3) loop of gp120 [15,16,17,18,19,20]. In our recent published paper, we further demonstrated that chimeric HPV16 VLPs presenting P18I10 and T20 peptides from HIV-1 envelope could induce HPV16 and HIV-1-specific humoral and T cell-mediated immunity in BALB/c mice [21]. The structural feature of BPV L1 capsid protein is similar to that of human papillomavirus type-16 (HPV16) [22]. Five of the HPV16 L1 proteins form a pentamer, also called capsomer, and 72 of the pentamers could self-assemble into single layer HPV16 L1 VLPs [23]. The early attempts to produce such chimeric BPV:HIV VLPs, the BEVS/IC system and ultracentrifugal purification method were the most commonly selected platforms [15,16,17,18]. Nevertheless, optimal production and purification conditions for such chimeric HPV:HIV (L1:P18I10) VLPs were not published.

The recombinant HPV16 L1 proteins were successfully produced in insect cells [24,25], yeast [26,27,28,29], bacterial [30,31,32,33], and plants [34,35]. The baculovirus expression vector and insect cell (BEVS/IC) system is the most commonly used platform for VLP production [36,37]. For example, the FDA-approved Cervarix vaccine consisting of HPV16/18 L1 VLPs relied on BEVS/IC for commercial large-scale production [38,39]. The early attempts to generate chimeric BPV L1:P18I10 VLPs also selected the BEVS/IC system [15,16,17,18]. The BEVS/IC system has advantages over the mammalian expression system to reach a high expression level of recombinant proteins that is comparable with bacteria and yeast expression systems [40]. The disadvantage of the BEVS/IC platform could attribute to the baculoviruses that must be inactivated or removed through extra downstream steps, such as chromatography [41]. Although the mammalian cell expression system provides a baculovirus-free purification condition, low expression level and high cost could be its major drawbacks [36]. Until now, optimum conditions of production HPV16 L1 proteins in the mammalian expression system were not well-developed. Therefore, we aimed to use 293F cells combined with cost-effective polyethylenimine (PEI), which could be a substitute for commercial transfection reagents, to reach an appreciable expression level of 293F cell-derived L1:P18I10 VLPs.

Ultracentrifugal approaches, such as sucrose cushion (SC) or cesium chloride (CsCl) density gradients, were widely used to isolate the HPV6 L1 VLPs previously because the large VLP mass (MW > 20,000 kDa) was separated to most of contaminants [22,25,26,31,33,34,35]. In many early studies, SC and CsCl ultracentrifugation were preferable to purify chimeric BPV:HIV (L1:P18I10) VLPs [15,16,17,18]. However, the conventional ultracentrifugation procedures were quite time-consuming and difficult for industrial scaling-up. A large proportion of target protein was lost during purification, and the recovery rate (~10%) was relatively low [41]. A substantial quantity and quality of VLP-based vaccine is necessary for biophysical characterization and downstream immunogenic test. Therefore, an industrial trend is observed from ultracentrifugation towards scalable chromatography [37,42]. The different physiological purification conditions, together with bioprocessing parameters, might vary overall purity, recovery, in vitro stability, and even immunogenicity of HPV:HIV (L1:P18I10) VLP products.

In this study, the HPV 16 L1 protein acted as the structural vaccine scaffold, and the P18I10 peptide was selected as HIV-1 immunogen and inserted into DE loop of HPV16 L1 protein. The immunodominant P18I10 CTL epitope comprising 10 amino acids (residues 311–320: RGPGRAFVTI) is derived from the third variable domain (V3) of the HIV-1 Env gp120 [43,44]. The HIV-1 P18I10 epitope were selected as a starting point and proof of concept experiment for the chimeric VLP-based HPV:HIV vaccine development platform. We first evaluated the level of L1:P18I10 protein and transfection efficiency by using the BEVS/IC and 293F expression systems. Then, ultracentrifugal and chromatographic purification methods were optimized through testing various parameters, such as pH and ionic strength of buffers. After purification, the recovery and purity of L1:P18I10 VLPs in each ultracentrifugal and chromatographic purification step were evaluated. In vitro stability test, molecular weight assessment and transmission electron microscope (TEM) were performed to determine the biophysical properties of L1:P18I10 VLPs. The purified L1:P18I10 proteins were further identified by immunoblotting probed with HPV16 L1 and HIV-1 V3 mAbs. Finally, immunogenicity of chromatography-purified L1:P18I10 VLPs in BALB/c mice was assessed by ELISA and ELISpot. Here, we demonstrated that 293F expression system combining with chromatography could be feasible and scalable approaches to engineer chimeric HPV:HIV (L1:P18I10) VLP-based vaccines for the future industrial manufacturing. This study provided a baseline of production and purification protocol that may be worthy to support the global efforts to develop novel chimeric VLP-based vaccines for controlling HPV and HIV-1 infections.

## 2. Results

### 2.1. Comparison of L1:P18I10 Proteins Production in BEVS/IC and 293F Expression Systems

**Figure 1:**
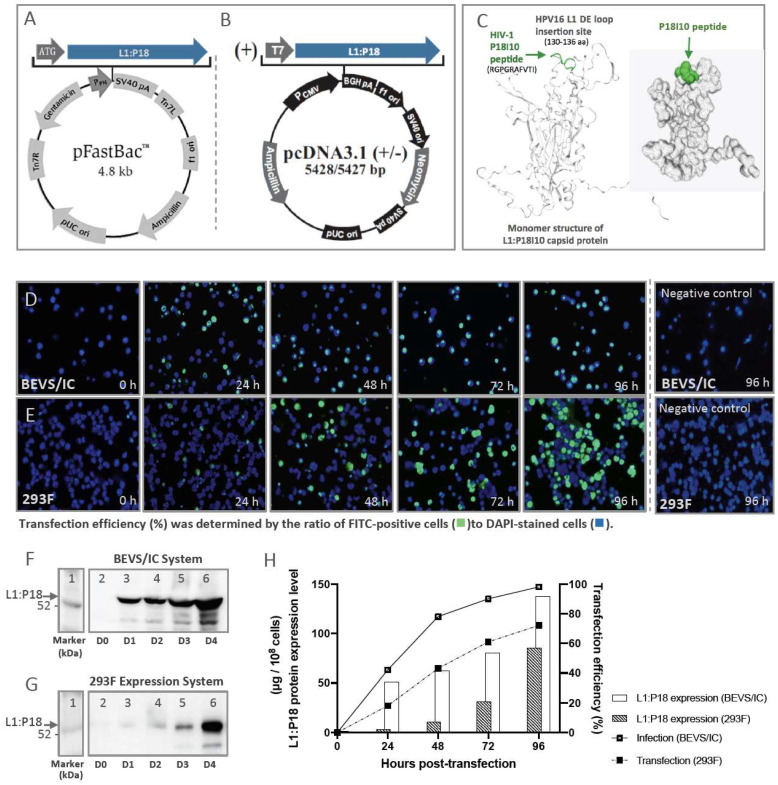
L1:P18I10 proteins production and transfection efficiency by using BEVS/IC or 293F expression systems. (**A**,**B**) The chimeric L1:P18I10 DNA coding sequence were cloned into pFastBac1 and pcDNA3.1+ vector for BEVS/IC or 293F expression systems, respectively. (**C**) The structural template of HPV16 L1 capsid (6bt3.1.I) and model-building of L1:P18I10 capsid protein was analyzed by using SWISS-model server. (**D**,**E**) Immunofluorescence staining of L1:P18I10 proteins produced from BEVS/IC and 293F systems. Sf9 cells (top panel) and 293F cells (bottom panel) were harvested in day 0 to day 4 post-transfection. Negative control Sf9 cells were not infected with recombinant baculovirus and harvested in 96 h. Negative control 293F cells were transfected with empty pcDNA3.1 plasmid DNA and harvested in 96 h post-transfection. Cells were probed with anti-HPV16 L1 mAb and detected with anti-mouse IgG-FITC (green channel). Cell nuclei were stained with DAPI (blue channel). Immunofluorescence images were merged by using Adobe Photoshop. (**F**,**G**) Western blot analysis of L1:P18I10 proteins produced from BEVS/IC and 293F systems. A total of 1 × 10^8^ Sf9 or 293F cells in day 0 to day 4 post-transfection were collected and analyzed by Western blot stained with anti-HPV16 L1 mAb. Lane 1: protein molecular weight marker; Lane 2: 0 h; Lane 3: 24 h; Lane 4: 48 h; Lane 5: 72 h; Lane 6: 96 h. (**H**) Comparison of transfection efficiency and L1:P18I10 protein expression level between BEVS/IC and 293F expression systems. The expression level of L1:P18I10 proteins was densitometrically quantified by Image Studio Lite 5x software. The HPV16 L1 proteins were used as a control for quantification. Transfection efficiency was determined by the ratio of FITC(green)-positive cells to DAPI(blue)-stained cells.

The chimeric L1:P18I10 DNA coding sequence was cloned into pFastBac1 and pcDNA3.1 plasmid DNA expression vector, respectively, as shown in Figure 1A,B. We aimed to compare the feasibility of the polyethylenimine (PEI)-mediated transfection using pcDNA3.1 vector in human 293F cells with that of the recombinant baculovirus-mediated transfection in insect Sf9 cells. The secondary structure of the chimeric L1:P18I10 capsid protein was predicted based on SWISS-modeling (Figure 1C). HPV16 major L1 capsid protein (6bt3.1.I) was identified as the structural template. Because L1:P18I10 capsid proteins could homogeneously arrange into T = 7 icosahedral particles with 72 pentamers [45], the P18I10 epitope should be theoretically exposed to the exterior DE loop of the L1 capsid protein in a high density (~360 copies) to induce epitope-specific immune responses.

We used immunofluorescence staining to determine the expression of L1:P18I10 proteins and evaluate the polyethylenimine (PEI) or baculovirus-mediated transfection efficiency from day 0 to day 4 (Figure 1D,E). The CAMVIR-1 monoclonal antibody was selected to recognize HPV16 L1 epitope (GFGAMDF, 230–236 aa) [46], and fluoresce-in-based dye FITC (green) was used as reporter. Transfection efficiency (%) was determined by the ratio of FITC-positive cells to DAPI (blue)-stained cells. Approximately 42% of the Sf9 cells were positively stained in the first day post-infection. Subsequently, the positively stained Sf9 cells increased sharply to 78% in day 2 and reached 98% in day 4 (Figure 1D). By contrast, L1 signals were detected in only around 18% of 293F cells in day 1 post-transfection, indicating that 36 h post-transfection might be optimal timing for endocytic uptake of the PEI-DNA complex into cells. FITC-positive 293F cells increased gradually from 43% to 61% in day 2 and day 3. Up to 72% of FITC-positive cells were obtained in day 4 (Figure 1E). Some irregular DAPI-stained cell nuclei were detected and could be attributed to the cytopathic effect caused by baculovirus or cytotoxicity resulted from PEI.

Since frequency and intensity of L1 signals detected by immunofluorescence did not directly correlate with L1:P18I10 protein expression level in the host cells, we further quantified L1:P18I10 capsid proteins by Western blot analysis (Figure 1F,G). L1:P18I10 proteins extracted from Sf9 cells were detected as a band in size of around 56 kDa (Figure 1F). Several lower bands in size of less than 52 kDa were detected and probably caused by proteolytic degradation or heterogeneous formation of L1:P18I10 proteins. In 293F cells, relatively weak L1 signals were detected from day 1 to day 3 post-transfection. However, the L1 signal was significantly enhanced in day 4 (Figure 1G). In both expression systems, the expression level of L1:P18I10 proteins observed by Western blot were consistent with that detected by immunofluorescence.

**Figure A1:**
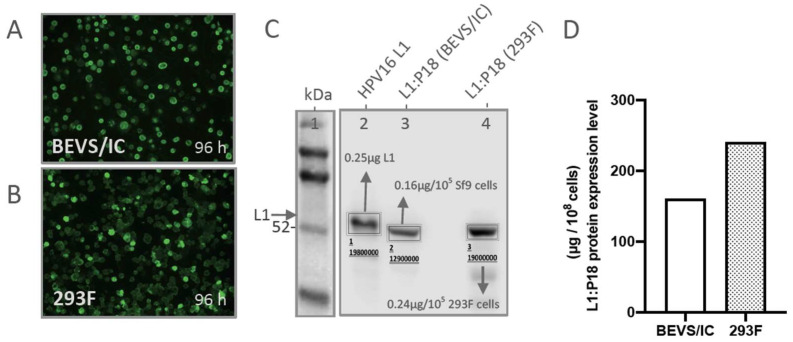
L1:P18I10 proteins production and transfection efficiency by using BEVS/IC or 293F expression systems. (**A**,**B**) Immunofluorescence staining of L1:P18I10 proteins produced from BEVS/IC and 293F systems. Sf9 cells (top panel) and 293F cells (bottom panel) were harvested in day 4 post-transfection. Cells were probed with anti-HPV16 L1 mAb and detected with anti-mouse IgG-FITC (green channel). (**C**) Quantification Western blot analysis of L1:P18I10 proteins produced from BEVS/IC and 293F systems. A total of 1 × 10^5^ Sf9 or 293F cells in day 4 post-transfection were collected and analyzed by Western blot stained with anti-HPV16 L1 mAb. The expression level of L1:P18I10 proteins was densitometrically quantified by Image Studio Lite 5x software. The HPV16 L1 proteins were used as a control for quantification. Lane 1: protein molecular weight marker; Lane 2: 0.25 µg HPV16 L1 protein; Lane 3: BEVS/IC-produced L1:P18 protein; Lane 4: 293F-produced L1:P18 protein. (**D**) Comparison of L1:P18I10 protein expression level between BEVS/IC and 293F expression systems. The expression level of L1:P18I10 proteins was densitometrically quantified by Image Studio Lite 5.x software. The HPV16 L1 proteins were used as a control for quantification.

As shown in Figure 1H, a comparison between BEVS/IC and 293F expression systems was made to correlate the transfection efficiency with corresponding L1:P18I10 protein expression level in overall pattern. Transfection efficiency (~72%) of PEI was lower than infection efficiency (~98%) of baculoviruses. The expression level of L1:P18I10 proteins using 293F expression system (85.39 µg per 1 × 10^8^ 293F cells) was approximately ~39% lower than BEVS/IC system (137.87 µg per 1 × 10^8^ Sf9 cells) in day 4 post-transfection. As shown in Figure A1A,B, transfection efficiency (~90%) of PEI could be comparable with infection efficiency (~98%) of baculoviruses. After quantification L1:P18I10 capsid proteins by Western blot analysis (Figure A1C), the expression level of L1:P18I10 proteins using 293F expression system (240 µg per 1 × 10^8^ 293F cells) could be higher than BEVS/IC system (160 µg per 1 × 10^8^ Sf9 cells) in day 4 post-transfection (Figure A1D). Therefore, 293F expression system could be an alternative method of BEVS/IC system to produce comparable L1:P18I10 proteins for downstream purification.

### 2.2. Optimization of L1:P18I10 VLP Purification Using Ultracentrifugal or Chromatographic Methods

#### 2.2.1. Optimization of Ultracentrifugal Purification Methods by Using BEVS/IC-Derived L1:P18I10 VLP

**Figure 2:**
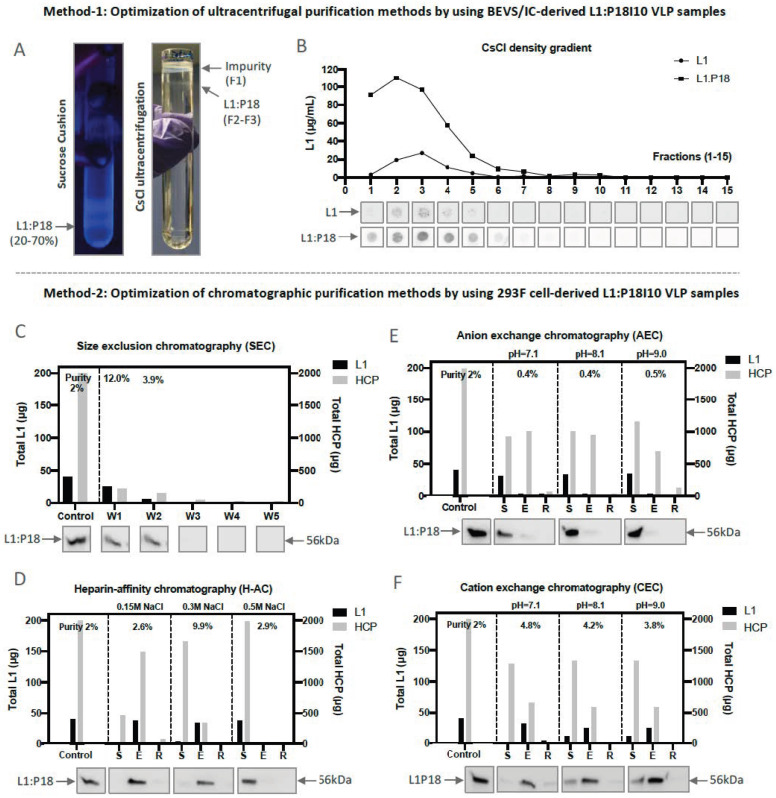
Optimization of L1:P18I10 VLPs purified by using ultracentrifugal or chromatographic methods. (**A**) Purification of L1:P18I10 VLPs using ultracentrifugal methods. L1:P18I10 VLPs were partially purified by a two-step SC (left) and, subsequently, fractionated by CsCl gradient (right). The concentrated L1:P18I10 VLPs were indicated by the arrows. (**B**) Detection profiles of L1:P18I10 VLPs in CsCl density gradient. The CsCl gradient was fractionated from the top of the tube (F1-F15, 400 μL per fraction). Fraction 1 corresponds to the top of the tube. The signal of L1:P18I10 VLPs in each fraction was detected by dot blot, using anti-HPV16 L1 mAb. The HPV16 L1 VLPs were used as a positive control. The peak of the line graph indicates the corresponded fraction in which VLPs were detected. (**C**) Optimization of SEC. (**D**) Optimization of H-AC. (**E**) Optimization of AEC. (**F**) Optimization of CEC. In each independent test, a total 2 mg of soluble 293F cell lysate containing around 2% of L1:P18I10 VLPs was loaded into the column. The flow-through (FT) collected from each purification step were loaded on SDS-PAGE gels, which were Coomassie-stained, and analyzed by Western blot, using HPV16 L1 mAb. The arrow indicates the molecular weight ~56 kDa of L1:P18I10 protein. The L1 and HCP were quantified by densitometric assay using Image Studio Lite 5.x software and represented in column charts. Purity (%) was determined by the ratio of L1 to HCP. Control: soluble cell lysate; W1-5: eluate collected from washing step; S: flow-through (FT) collected from sample loading; E: eluate collected from elution; R: FT collected from 2M NaCl regeneration step.

**Figure A2:**
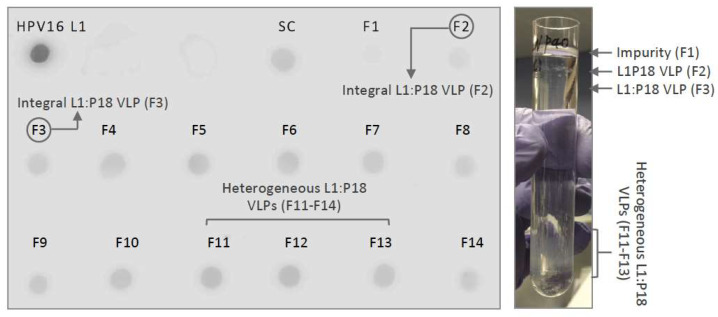
Detection of BEVS/IC-derived L1:P18I10 VLPs in CsCl density gradient. BEVS/IC-derived L1:P18I10 VLPs were partially purified by a two-step sucrose cushion (SC) and subsequently fractionated by CsCl gradient. The CsCl gradient was fractionated from the top of the tube (F1-F15, 400 μL per fraction). Fraction 1 corresponds to the top of the tube. The signal of L1:P18I10 VLPs in each fraction was detected by dot blot, using anti-HPV16 L1 mAb. The HPV16 L1 VLPs were used as a positive control. CsCl-purified VLPs could be heterogeneous in size because of the broken particles and presented as multiple bands.

Based on the results of previous studies, purification of papilloma virus VLPs was heavily relied on BEVS/IC and ultracentrifugal techniques [22,25,26,33,34,47,48,49,50,51]. Therefore, we used a two-step (20% and 70%) sucrose cushion (SC) as a preliminary capturing step and caesium chloride (CsCl) density gradient as an intermediated step to concentrate and purify L1:P18I10 VLPs produced from BEVS/IC systems. The L1:P18I10 VLPs formed a distinctive band under UV light at the interface layer between 20% and 70% sucrose (Figure 2A, left). Because density of impurities was reported to be lower than VLPs during CsCl ultracentrifugation [48], we found that L1:P18I10 VLPs appeared in a single but a bit diffuse band under impurities to the top of CsCl tube (Figure 2A, right). In some cases, CsCl-purified VLPs could be heterogeneous in size because of the broken particles and presented as multiple bands (Figure A2). Since it was known that HPV16 L1 capsid proteins is visualized at a density of approximately 1.29 g/cm^3^ in the CsCl gradient [35], we used commercial HPV16 L1 VLPs as a control to determine the major peak of L1:P18I10 VLPs (Figure 2B). We observed that L1:P18I10 VLPs could be quite homogeneous in density with wild-type HPV16 L1 VLPs.

#### 2.2.2. Optimization of Chromatographic Purification Methods by Using 293F-Derived L1:P18I10 VLP

Since the HiLoad pump is difficult to perform linear ionic strength or pH gradients, we performed one-step gradient elution for chromatographic method development when starting with our unknown L1:P18I10 VLP samples. The ionic strength or pH parameters obtained can then serve as a base from which to optimize the separation of L1:P18I190 VLPs. A total 2 mg of host cellular proteins (HCPs) containing approximately 2% of L1:P18I10 VLPs produced from 293F expression system was loaded into the size exclusion chromatography (SEC), heparin affinity chromatography (H-AC), and ion exchange chromatography (IEX) columns, respectively (Figure 2C–F). As shown in Figure 2C, overall purity of L1:P18I10 VLP was increased from 2% to 12% (6-fold) (80.7% L1:P18I10 VLP recovery and 75.8% HCP removal) after purification in flow-through mode using a layered-bead size exclusion medium (>MW 700 kDa) [52]. As shown in Figure 2D, L1:P18I10 VLP purity could increase from 2% to 9.9% (5-fold) (89% L1:P18I10 VLPs binding capacity, 85% L1:P18I10 VLPs recovery and 83% HCP removal) using a heparin resin in optimal ionic strength of 300 mM NaCl.

Although an application note of the general electric (GE) company indicated that disassembled HPV16 L1 monomers can bind to anion exchange chromatography media (AEC) at pH 8.5 [52], we observed that that almost all of reducing agent dithiothreitol (DTT)-treated L1:P18I10 proteins were not bound to AEC in a range of pH (7.1–9.0) (Figure 2E). We suspected that maximal disassembly of L1:P18I10 VLPs might required not only reducing agents but also other denaturing factors [53,54]. By contrast, we found that L1:P18I10 VLPs could bind to cation exchange chromatography media (CEC) at a wider range of pH (7.1–9.0). As shown in Figure 2F, purity of L1:P18I10 VLPs slightly increased from 2% to 4.8% (2.5-fold) (95% L1:P18I10 VLPs binding capacity, 79% L1:P18I10 VLPs recovery and 67% HCP removal) using negative-charged resins at an optimal pH 7.1. This pattern is the same as prior study indicating that HPV16 L1 VLPs could bind to CEC at pH 7.2 in a native form [35]. HPV-16 L1 proteins have an isoelcetric point (pI) of 7.95 and carry positive charge of +2.98 at pH 7.4 [55]. Although L1:P18I10 protein was predicted to have the similar pI of 8.2 to wild-type HPV16 L1 by using the on-line pI calculator, we deduced that L1:P18I10 VLPs might authentically have a higher pI of around 10.

### 2.3. Comparison of L1:P18I10 VLP Purification Using Ultracentrifugation or Chromatography

**Figure 3:**
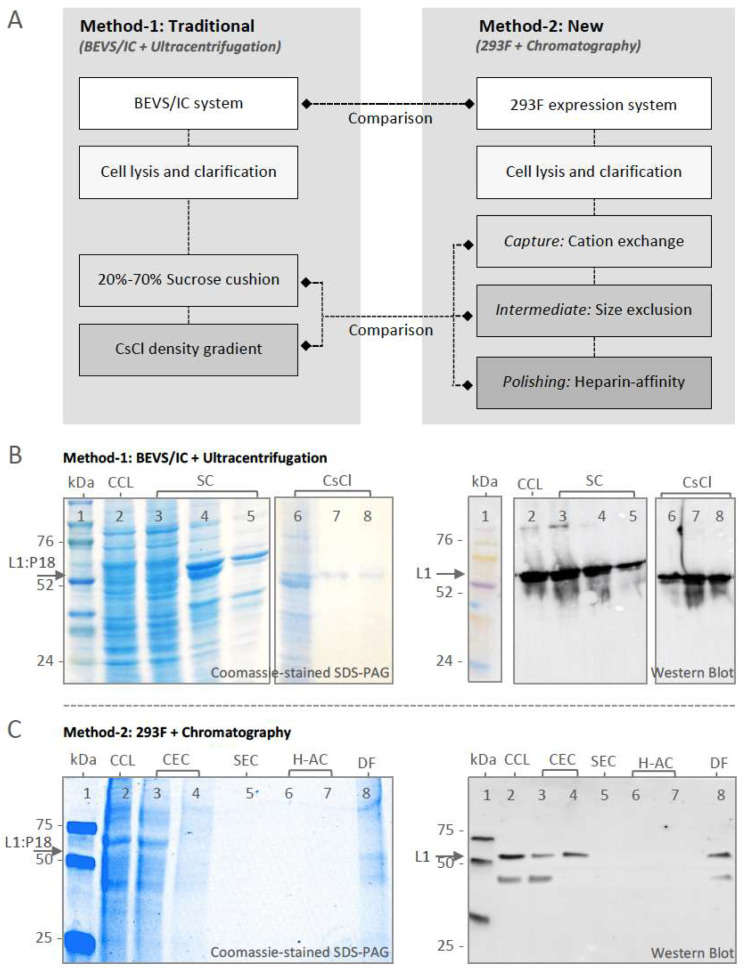
Purification and characterization of L1:P18I10 VLPs. (**A**) Schematic process flowchart of L1:P18I10 VLP purification. The BEVS/IC expression system and ultracentrifugation (traditional method-1) were served as standard control methods in comparison with 293F expression system and chromatography (new method-2). (**B**) Characterization of BEVS/IC-derived L1:P18I10 VLPs purified by using SC and CsCl ultracentrifugation. L1:P18I10 VLP samples collected from different layers of SC and fractionated from CsCl gradients were analyzed by Coomassie-stained SDS-PAGE (left panel) and Western blot probed with HPV16 L1 mAb (right panel). The arrow indicates the molecular weight ~56 kDa of L1:P18I10 protein. Lane 1: protein molecular weight marker; Lane 2: clarified cell lysate (CCL); Lane 3: 0–20% interface of SC; Lane 4: 20–70% interface of SC; Lane 5: 70% tube bottom of SC; Lane 6: fraction-1 of CsCl; Lane 7: fraction-2 of CsCl; Lane 8: fraction-3 of CsCl. (**C**) Characterization of 293F-derived L1:P18I10 VLPs purified by using chromatography. L1:P18I10 VLPs produced in 293F cells underwent CEC, SEC and H-AC chromatography. Flow-though (FT) collected from different chromatographic purification steps were analyzed by Coomassie-stained SDS-PAGE gel (left panel) and Western blot probed with HPV16 L1 mAb (right panel). The arrow indicates the molecular weight ~56 kDa of L1:P18I10 protein. Lane 1: protein molecular weight marker; Lane 2: CCL; Lane 3: FT from CEC sample loading; Lane 4: CEC eluate; Lane 5: SEC FT; Lane 6: FT from H-AC sample loading; Lane 7: H-AC eluate; Lane 8: 10-fold diafiltration.

By following previous studies [34,35,48], L1:P18I10 VLPs produced from BEVS/IC expression system were purified through a two-step SC (20% and 70%) followed by a CsCl density gradient (Figure 3A, left panel). As shown in Figure 3B, samples collected from different layers of SC and fractionated from the CsCl tube were analyzed by Coomassie-stained SDS-PAGE and Western blot. Most of the unwanted HCPs were retained at the top layer of 20% SC (Figure 3B, lane 3). The concentrates collected from the interface between 20 and 70% SC and the bottom of 70% SC were partially purified L1:P18I10 VLPs (Figure 3B, lane 4 and 5). The fraction-1 collected from the top of the CsCl gradient contained most of impurities (Figure 3B, lane 6). Pure L1:P18I10 VLPs were detected in fraction-2 and 3 (Figure 3B, lane 7 and 8). Total L1 and HCPs were quantified by band intensity from the Western blot and bovine serum albumin (BCA) assay, respectively, and shown in Table 1. Approximately 99% of contaminants were removed, 11% of L1:P18I10 proteins were recovered, and the purity of L1:P18I10 VLPs was increased from 4% to 99% (25-fold) after the SC and CsCl ultracentrifugation. These results corresponded to those reported in earlier studies, which provided a low assumption of VLP recovery of around 10% [48].

Based on optimized chromatographic parameters obtained from the previous section, we designed a capture, intermediate purification and polishing (CiPP) chromatographic strategy to purify 293F cell-derived L1:P18I10 VLPs in flow-through mode using CEC, SEC, and H-AC (Figure 3A, right panel). Flow-though (FT) collected from each chromatographic step was analyzed by Coomassie-stained SDS-PAGE and Western blot (Figure 3C). Most of HCPs (~65%) were removed by CEC (Figure 3C, lane 3). Only ~0.64 mg HCPs, including 21.5 μg L1:P18I10 VLPs, were captured by CEC (Figure 3C, lane 4). Since eluate collected from CEC was diluted 4-fold before loading on SEC, HCP and L1 signals were too weak to be shown on SDS-PAGE and Western bot (Figure 3C, lane 5–7). As shown in Figure 3C, lane 8, the eluate collected from H-AC were finally concentrated 10-fold through diafiltration. The SDS-PAGE gel provided a visual image of the purified L1:P18I10 proteins and the removal of HCPs. A lower band in a size of less than 50 kDa was detected in SDS-PAGE and Western blot analysis. It probably caused by heterogonous L1:P18I10 proteins or proteolytic degradation. The L1 and HCPs were further quantified by densitometric assay of Western blot and BCA assay, respectively, and presented in Table 1. Approximately 98% of HCP impurities were removed, and the purity of L1:P18I10 VLPs was increased from 2% to 76% (38-fold). Compared with 11% recovery of L1:P18I10 VLPs by using ultracentrifugal approaches, chromatographic methods effectively improved recovery of L1:P18I10 VLPs to 56% (approximately 6-fold) and might be available for scaling up.

### 2.4. In Vitro Stability and Self-Assembly of Ultracentrifugation- and Chromatography-Purified L1:P18I10 VLPs

**Figure 4:**
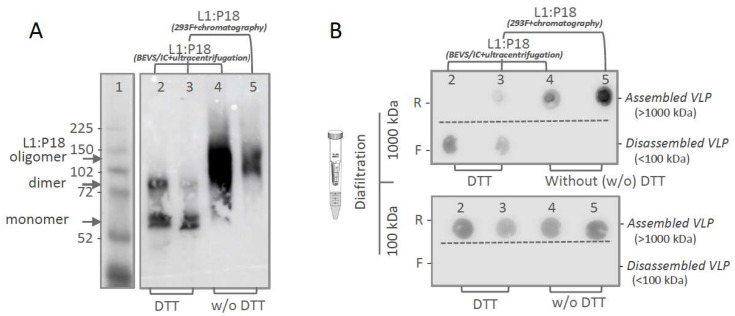
In vitro stability and self-assembly of purified L1:P18I10 VLPs. (**A**) Disulfide cross-linking of L1:P18I10 proteins in non-reducing SDS-PAGE. Purified L1:P18I10 VLPs were mixed with Laemmli sample buffer in the presence or absence of DTT, respectively, and analyzed by non-reducing SDS-PAGE. The position of L1:P18I10 monomer (56 kDa), dimer (112 kDa) and oligomer (112~224 kDa) are indicated by the arrow on the right. Lane 1: protein molecular weight marker; Lane 2: ultracentrifugation-purified L1:P18I10 treated with DTT; Lane 3: chromatography-purified L1:P18I10 treated with DTT; Lane 4: ultracentrifugation-purified L1:P18I10; Lane 5: chromatography-purified L1:P18I10. (**B**) Molecular mass analysis of L1:P18I10 VLPs. L1:P18I10 VLPs purified from both methods were treated (lane 2 and 3) or un-treated (lane 4 and 5) with DTT and filtered out through 1000 kDa or 100 kDa MWCO centrifugal filter devices. Retentates (R) were collected from filter device sample reservoirs, while filtrates (F) were collected at the bottom of centrifuge tubes. The L1 signal was detected by using dot blot probed with anti-HPV16 L1 mAb.

In order to assess in vitro stability of purified L1:P18I10 VLPs, we performed non-reducing SDS-PAGE to evaluate disulfide cross-linking of L1:P18I10 capsid proteins (Figure 4A). It is known that pH, ionic strength, temperature [56], and redox environment all correlate with disulfide bonds of HPV16 L1 capsid proteins [53]. HPV16 L1 VLPs tend to self-assemble at low pH and high ionic strength. On the contrary, reducing agents such as dithiothreitol (DTT) could significantly disassemble HPV16 L1 VLPs into monomers [52]. In the presence of reducing agent DTT, ~50% of ultracentrifugation-purified L1:P18I10 and ~80% chromatography-purified L1:P18I10 proteins appeared monomeric structure (Figure 4A, lane 2 and 3). A small proportion of L1:P18I10 dimers was also detected. In the absence of DTT, above 99% of L1:P18I10 proteins purified from both methods was di-sulfide bonded into larger oligomers with predicted molecular weight (MW) of ~110 to 280 kDa (Figure 4A, lane 4 and 5). L1:P18I10 oligomers were not completely resolved and did not migrate to a single band and appeared to be heterogeneous in size. These results indicated that in vitro stability of L1:P18I10 VLPs purified from both methods presented a similar pattern under the same pH, ionic strength, and thermal condition.

In order to demonstrate that purified L1:P18I10 proteins from both methods are able to self-assemble to icosahedral particles, we further performed molecular mass assessment. As shown in Figure 4B, top, purified L1:P18I10 VLP samples with or without DTT treatment were filtered out through 1000 kDa MWCO diafiltration devices individually. The L1 monomers (55 kDa) and oligomers (110~280 kDa) were expected to pass through an ultrafiltration membrane retaining the integral L1:P18I10 VLPs (MW > 20,000 kDa). In the presence of DTT, L1:P18I10 proteins purified from both methods were disulfide reduced and detected in filtrates. In the absence of DTT, L1:P18I10 proteins formed large particles (>1000 kDa) and preserved in retentates. The pattern was well in line with the data rep-resented in non-reducing SDS-PAGE. Although most of the L1:P18I10 proteins from both methods treated with DTT were showed in monomer bands in the non-reducing SDS-PAGE (Figure 4A, lane 2 and 3), reduced L1:P18I10 proteins were not filtered out through 100 kDa ultrafiltration membranes (Figure 4B, lane 2 and 3). These results indicated that maximal disassembly of the L1:P18I10 VLPs might require not only the reduction in disulfide bonds but also other denaturing factors [53,54]. Additionally, L1:P18I10 proteins purified from both methods were capable of self-assembling to larger particles without reducing agent DTT treatment.

### 2.5. Morphological Characterization of L1:P18I10 VLPs Purified by Using Ultracentrifugal or Chromatographic Methods

**Figure 5:**
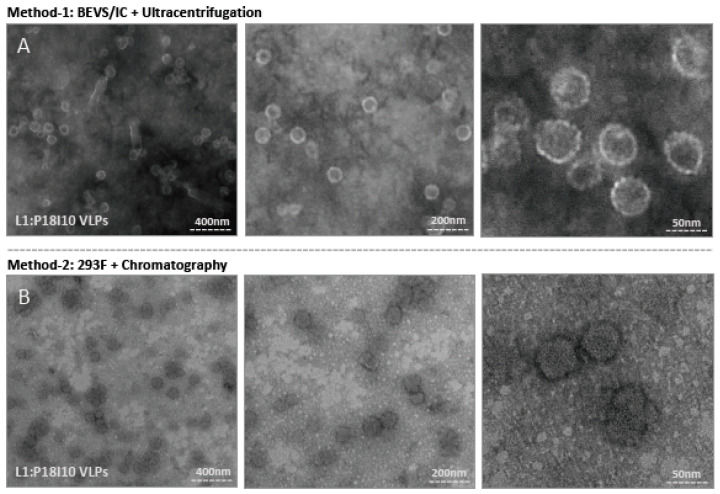
Electron micrographs of purified L1:P18I10 VLPs. (**A**) Morphology of ultracentrifugation-purified L1:P18I10 VLPs. L1:P18I10 VLPs were equilibrated with PBS, absorbed on UV-charged carbon-coated copper grids, and negatively stained with 2% PTA. (**B**) Morphology of chromatography-purified L1:P18I10 VLPs. L1:P18I10 VLPs were equilibrated with Tris-HCl, absorbed on UV-charged carbon-coated copper grids, and negatively stained with 2% uranyl acetate. Images were acquired under transmission electron microscopy Tecnai Spirit 120 kV. The bar represents 50 nm at magnification SA270K (left panel), 200 nm at magnification SA59000 (middle panel) and 400 nm at magnification SA529500 (right panel).

Transmission electron microscopy (TEM) was used to examine the morphologic conformation of purified L1:P18I10 VLPs. As shown in Figure 5A, the morphology of L1:P18I10 VLPs collected from the CsCl gradient was presented in diameter of 50–60 nm and similar to HPV16 L1 VLPs produced by BEVS/IC systems described in previous studies [22]. These particles were stained centrally, indicating DNA was not encapsulated. However, tubular structures of baculoviruses in length of 230–385 nm and diameter of 40–60 nm were observed at lower magnification (Figure 5A, left). It meant that ultracentrifugal approaches were difficult to remove remaining baculovirus generated by BEVS/IC systems. The structure of L1:P18I10 VLPs purified by ultracentrifugation was more spherical and regular, compared with that purified by using chromatographic method. As described in prior studies, ultracentrifugation seemed to provide a more gentle way for VLP purification [48].

The chromatography-purified L1:P18I10 proteins can also in vitro self-assemble into VLPs in a diameter of around 50–60 nm (Figure 5B). The morphology of these L1:P18I10 VLPs was a bit heterogeneous and irregular in shape with some loss of icosahedral structure at higher magnification (Figure 5B, right). We observed many smaller or broken particles less than 20 nm, which might be caused by flow-though pressures of chromatography. In our recent study [57], we obtained good quality and resolution of electron micrographs when yeast- and baculovirus-derived L1:P18I10 VLPs (Figure 5A) were equilibrated in PBS and negative-stained with phosphotungstic acid (PTA). Because we used the different VLP production and purification system in this study, chromatography-purified L1:P18I10 were equilibrated with Tris-HCl and negative-stained with uranyl acetate. Although the electron micrographs could be improved, we could still observe a clear pattern that most of the chromatography-purified L1:P18I10 capsid protein could self-assemble into morphological VLPs under TEM screen (Figure 5B, left panel). Basically, HPV L1 VLPs are protected against aggregation in high salt conditions (~0.5 M NaCl) [58]. The aggregation of chromatography-purified L1:P18I10 VLPs in low salt buffer (~137 mM NaCl) was detectable (Figure 5B, left). From these results, we concluded that both ultracentrifugal and chromatographic methods did not affect the capacity of L1:P18I10 proteins self-assemble into VLPs, but ultracentrifugation seems to be more favorable for integral VLPs formation.

### 2.6. Quantification and Epitope-Characterization of Ultracentrifugation- and Chromatography- Purified L1:P18I10 VLPs

**Figure 6:**
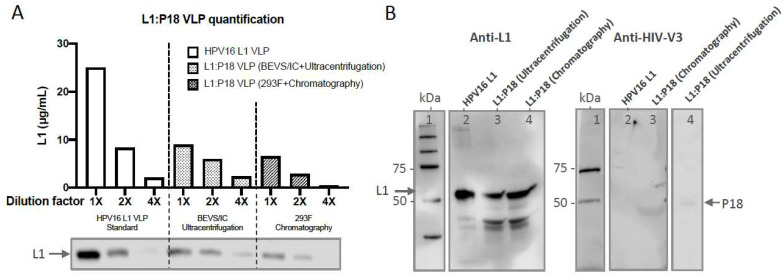
Characterization of ultracentrifugation- and chromatography-purified L1:P18I10 VLPs. (**A**) Quantification of L1:P18I10 VLPs. The concentration of purified L1:P18I10 VLPs by using both methods was extrapolated from a standard curve of HPV16 L1 VLPs plotting dilution factors versus concentration. The L1 band intensity from the Western blot was quantified by densitometric assay using Image Studio Lite 5.x software and illustrated in a bar diagram. (**B**) HPV-16 and HIV-1 epitope detection of L1:P18I10 VLPs. Purified L1:P18I10 VLPs were analyzed by Western blot, using anti-HPV16 L1 (left) and anti-HIV1 V3 mAb (right). The recombinant HPV16 L1 VLPs were used as a control. The position of the L1 (55 kDa) and L1:P18I10 (56 kDa) proteins are indicated by the arrow on the left and right, respectively. Lane 1: protein molecular weight marker; Lane 2: HPV16 L1 VLP; Lane 3: BEVS/IC-derived and ultracentrifugation-purified L1:P18I10 VLP; Lane 4: 293F-derived and chromatography-purified L1:P18I10 VLP.

To confirm whether HIV-1 P18I10 epitopes were expressed on chimeric HPV-16 L1 capsid proteins, we first extrapolated actual concentration of purified L1:P18I10 VLPs from a standard curve of commercial HPV16 L1 VLPs (Figure 6A). Equal amounts (0.2 µg) of ultracentrifugation or chromatography-purified L1:P18I10 VLPs were analyzed by Western blot using anti-HPV16 L1 and anti-HIV1 V3 mAbs. We selected a well-known HPV16 L1 monoclonal antibody, designated CAMVIR-1, to recognize the highly conserved epitope (GFGAMDF, aa 230–236) [46,59]. A HIV-1 gp120 V3 loop monoclonal antibody targeting the CTL epitope (RIQRGPGRAFVTIGK, aa 308–322) was used to detect the P18I10 peptide (RGPGRAFVTI, aa 311–320) [60]. Western blot probed with anti-HPV16 L1 mAb showed bands of around 55 and 56 kDa corresponding to wild-type HPV16 L1 VLPs and purified L1:P18I10 VLPs, respectively, (Figure 6B, left). By contrast, no P18I10 signal was detected in the lane of HPV16 L1 VLPs using anti-V3 antibodies (Figure 6B, right). Anti-HIV-1 gp120 V3 loop antibody recognized whole HIV-1 V3 loop rather than P18I10 epitope. Consequently, the overall anti-V3 signal was lower (Figure 6B, right). The results demonstrated that conformational and sequential HIV-1 P18I10 epitopes were presented in HPV16 L1 protein sequences.

### 2.7. Evaluation of HPV16- and HIV-1-Specific Humoral and Cellular Immune Responses Induced by 293F Cell-Derived and Chromatographic-Purified L1:P18I10 VLPs

**Figure 7:**
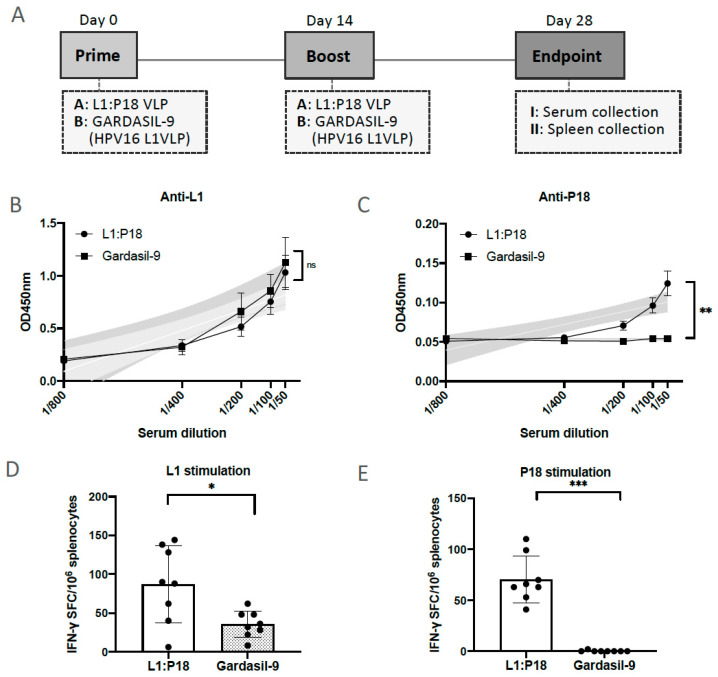
Induction in HPV16 and HIV1-specific antibodies and T-cell responses after L1:P18I10 VLP immunization in BALB/c mice. (**A**) Immunization schedule. Eight mice (male *n* = 4 and female *n* = 4 per group) in each group were immunized intramuscularly (i.m.) twice with either 10 μg of L1:P18I10 VLPs or 10 μg of Gardasil-9 (HPV16 L1 VLPs) vaccines. The homologous prime-boost interval was 2 weeks. The end point of this trial was on day 28. Sera and spleens were collected for ELISA and ELISpot assays, respectively. (**B**,**C**) L1 and P18I10-specific antibodies induced by L1:P18I10 VLPs. ELISA assay was performed to analyze anti-HPV16 L1 and anti-HIV1 P18I10 IgG induced by L1:P18I10 VLPs or Gardasil-9 in BALB/c mice. One group of mouse was immunized with only PBS buffer as a naïve group (negative control, without VLP) to set up the cutpoint. Simple linear regression test was carried out to compare the line difference between two groups. ns not significant; ** *p* < 0.01. (**D**,**E**) L1 and P18I10-specific T-cell responses induced by L1:P18I10 VLPs. IFN-γ ELISpot was performed to measure the frequency of IFN-γ secreting splenocytes after stimulation with HPV16 L1 VLP and P18I10 peptide induced by L1:P18I10 VLPs or Gardasil-9 in BALB/c mice. Data are shown as median ± S.D. Unpaired T test was performed to compare differences between groups. ns not significant; * *p* < 0.05; *** *p* < 0.001.

To evaluate whether chimeric L1:P18I10 VLPs induce HPV-16 L1 and HIV-1 P18I10-specific humoral and cell-mediated immune responses in BALB/c mice, the immunization schedule was designed as shown in Figure 7A. The chimeric L1:P18I10 VLPs purified from chromatography were administered in a homologous prime-boost regimen [11]. Since prior studies demonstrated that VLP-induced immunogenicity following mucosal administration was generally weaker than following systemic administration, mice were immunized intramuscularly with one sixth of Gardasil-9 HPV16 L1 VLP dose [18,61]. The aluminum hydroxyphosphate sulfate adjuvant of chimeric L1:P18I10 VLPs was adjusted to the same formulation (225 μg per each 0.5 mL dose) as Gardasil-9. The L1:P18I10 VLP-induced IgG antibodies in mice sera were measured by ELISA coated with HPV16 L1 VLPs or P18I10 peptides, respectively. As shown in Figure 7B, both HPV16 L1 VLPs and L1:P18I10 VLPs coated on the ELISA plate were recognized by the HPV16 L1 VLP- and L1:P18I10 VLP-induced L1 IgG antibodies in mice sera. We performed the linear regression analysis to compare the slope of each serum dilution line. The data revealed that anti-L1 IgG induced by chimeric L1:P18I10 VLPs was not different from HPV16 L1 VLPs (*p* = 0.6409). Moreover, the L1:P18I10 VLP-induced IgG in mice sera was able to bind L1:P18I10 VLPs, but not HPV16 L1 VLPs (Figure 7C). After the linear regression analysis, the differences of HIV-1 P18I10 epitope-binding antibody specificity between L1:P18I10 and HPV16 L1 were extremely different (*p* < 0.01%). One group of mouse was immunized with only PBS buffer as a naïve group (negative control, without VLP) to set up the cutpoint. Our immunogenicity data revealed that L1:P18I10 VLPs induced high anti-HPV16 L1, but lower anti-HIV-1 P18I10 antibodies (around 0.15 at MRD 1:50). After statistical analysis, the OD value for P18I10 induced IgG was significant from the naive PBS group. Therefore, we thought there is no pre-existing antibody against P18I10. Many other critical factors that could affect the immunogenicity of L1:P18I10 VLPs should be also considered, such as immunogen insertion site among different loops of VLPs, dose, prime-boost intervals, and administration route, etc. Overall, these results suggested that chimeric L1:P18I10 VLP-immunized mice produced almost the same level of anti-L1 IgG as Gardasil-9-immunized mice, and also elicited low anti-P18I10 binding antibodies.

To assess whether L1:P18I10 VLPs can induce HPV16- and HIV-1-specific cellular immune responses in vivo, splenocytes were collected and frequency of IFN-γ secreting splenocytes after HPV16 L1 VLP and P18I10 peptide stimulation was measured by IFN-γ ELISPOT assay. As shown in Figure 7D, differences in L1-specific IFN-γ secreting splenocytes were significant (*p* = 0.0132) between L1:P18I10 VLP and Gardasil-9 immunization groups. The difference might be attributed to the unspecific adjuvanticity of L1:P18I10 VLPs according our modified formulation. After unpaired *t* test analysis, an extremely higher frequency of IFN-γ secreting splenocytes was observed in mice homologously immunized twice with chimeric L1:P18I10 VLPs, as compared to mice receiving HPV16 Gardasil-9 vaccines in response to P18I10 peptide-stimulated splenocytes (*p* = 0.0002) (Figure 7E). These results demonstrated that chimeric L1:P18I10 VLP-immunized mice might be capable of producing significant P18I10-specific IFN-γ secreting splenocytes compared to Gardasil-9 control mice.

## 3. Discussion

The development of an affordable, safe, and effective preventive vaccine against HPV and HIV is still an urgent need. The capacity of production and purification system to engineer preparative expression level, purity, and yield (recovery) of chimeric HPV:HIV (L1:P18I10) VLPs may facilitate the development of VLP-based vaccine against both viruses. In this study, (i) we demonstrated that the 293F expression system could be an alternative of BEVS/IC system to produce comparable expression level of L1:P18I10 VLPs for downstream purification; (ii) the chromatographic purification method could significantly increased L1:P18I10 VLP recovery (56%) approximately 6-fold higher than ultracentrifugal approaches (11%); (iii) both ultracentrifugation and chromatography-purified L1:P18I10 VLPs shared similar biophysical properties in vitro: stability, in vitro self-assembly and morphology. However, ultracentrifugation provided a milder purification condition for integral L1:P18I10 VLPs formation; (iv) both ultracentrifugation and chromatography-purified L1:P18I10 VLPs could be characterized by HPV16 L1 and HIV-1 V3 (P18I10) mAbs; (v) chromatography-purified L1:P18I10 VLPs were immunogenic after BALB/c mice immunization. We anticipated that this scalable chromatography-based purification procedures will reduce the time, cost, and labor involved in industrial-scale manufacturing of VLP-based vaccines. This work contributes towards developing an alternative platform for production and purification of a bivalent VLP-based vaccine against HPV and HIV-1, which is urgently needed in developing and developed countries.

The P18I10 peptides derived from HIV-1 gp120 V3 loop are presented in HIV-infected cells by major histocompatibility complex (MHC-I) class I molecules [44]. CD8+, cytotoxic T lymphocytes (CTL), could recognize MHC-I restricted P18I10 peptides and secreting a variety of cytokines such as IFN-γ to eliminate HIV-infected cells [62,63]. In our previous study, we demonstrated that priming with recombinant *Mycobacterium bovis* Bacillus Calmette-Guérin (rBCG) expressing HIVA immunogen and boosting with recombinant viral vector MVA.HIVA was safe and elicited HIV-1-specific T-cell immune responses in BALB/c mice [64,65,66]. The HIVA immunogen, designed by Dr. Tomas Hanke, is composed of the full-length HIV-1 Gag protein combined with multiple CTL epitopes including P18I10 epitopes at the C-terminus [67]. In our recent publication, we demonstrated that our chimeric HPV:HIV (L1:P18I10) VLPs could induce HIV-specific T-cell immune responses in BALB/c mice after splenocytes stimulation with P18I10 peptide. In addition, BCG.HIVA prime and L1:P18I10 VLP boost elicited highest magnitude of IFN-γ producing splenocytes in comparison with L1:P18I10 VLPs homologous prime-boost in BALB/c mice [21]. These finding supported further development of HIV-1 vaccines based on rBCG and chimeric HPV:HIV VLPs.

A comparison of the expression level of L1:P18I10 proteins between BEVS/IC and 293F expression systems is not always straightforward, since production is also affected by complexity of VLPs and different cell culture conditions [36]. For instance, the low expression level and production of HPV L1 proteins using the BEVS/IC system was observed in certain HPV genotypes [47]. Depend on various types of HPV VLPs, the BEVS/IC system might reach a wide range of the VLP expression level between 0.2 mg/L and 125 mg/L [37]. In the case of licensed HPV vaccine manufacturing, the expression level of yeast-derived HPV16 L1 VLPs (Gardasil-4 HPV vaccine) was estimated to be 29 mg/L. The expression level of BEVS/IC-derived HPV16 L1 VLPs (Cervarix HPV vaccine) was around 40 mg/L [68]. Our data revealed that the amount of L1:P18I10 VLPs produced from 1 × 10^8^ Sf9 cells in 80 mL Grace’s insect/TNM-FH medium was around 137.87 µg. Therefore, the overall yield per unit culture volume (mg/L) of our BEVS/IC-derived L1:P18I10 VLPs was calculated to be 1.72 mg/L. In our laboratory, the Sf9 cell density at 96 h of harvest could only reach approximately 1.2 to 1.5 × 10^6^ cells/mL. This pattern match Merck’s application note indicating that the Sf9 cell density could expand from 1.0–1.2 × 10^6^ cells/mL to 1.5–2 × 10^6^ cells/mL through shake flask cultures to a bioreactor [41]. However, some studies reported that the density of Sf9 cells could reach over 10 × 10^6^ cells/mL by using fed-batch bioreactors under tightly monitored culture conditions for large-scale manufacturing production [37]. Therefore, the relatively lower expression level of our BEVS/IC-derived L1:P18I10 proteins might be attributed to laboratorial cell culture conditions, compared to fed-batch bioreactors in optimal conditions.

The BEVS/IC system was widely used in the pharmaceutical industry. Although both BEVS/IC and mammalian systems have post-translational modifications (PTM), the BEVS/IC system could only perform simpler glycosylation PTM, which is not in favor of enveloped VLP production [69]. Another crucial challenge of the BEVS/IC system is co-production of enveloped baculoviruses. This biophysical feature of baculoviruses may face purification hurdles if the VLP is also enveloped, such as Influenza and HIV-1 VLPs. Because the baculovirus itself has strong adjuvant properties, it might elicit synergistic humoral and CTL responses and interfere in the immunogenicity of target VLPs [70]. The remaining baculoviruses after ultracentrifugal purification methods might negatively affect the immunogenicity of L1:P18I10 VLPs. Therefore, purified L1:P18I10 VLPs should undergo baculovirus inactivation to eradicate the potential infectivity [71] or baculovirus removal through extra chromatographic steps. For instance, the ion exchange chromatography was shown to remove 10^2^ to 10^5^ baculovirus particles during VLP purification [41].

According to pre-studies, the expression level of recombinant proteins produced by using PEI-mediated transfection in 293E cells is around 22–50 mg/L [72,73,74]. Our results revealed that the amount of L1:P18I10 VLPs produced from 1.0 × 10^8^ 293F cells in 30 mL FreeStyle 293 expression medium was 85.39 µg. Thus, the overall yield per unit culture volume of 293F-derived L1:P18I10 proteins was calculated to be 2.85 mg/L. Since mammalian cells tend to be lower VLP-producers [36], the overall L1:P18I10 protein expression level using 293F expression system (85.39 µg per 1 × 10^8^ 293F cells) was lower than BEVS/IC system (137.87 µg per 1 × 10^8^ Sf9 cells). However, the 293F cells in shake flask suspension cultures can grow until a defined density of 3.0 to 3.6 × 10^6^ cells/mL, compared to Sf9 cell density of 1.2 to 1.5 × 10^6^ cells/mL. When we changed the measure of the L1:P18I10 protein expression level (yield) from weight per unit cell (µg/1 × 10^8^ cells) to weight per unit culture volume (mg/L), the overall L1:P18I10 protein yield using 293F expression system (2.85 mg/L) was higher than BEVS/IC system (1.72 mg/L). Therefore, we could re-clarify that the 293F expression system is capable of reaching an overall level of L1:P18I10 protein expression that could be comparable with BEVS/IC system.

Although 293F cells provide a baculovirus-free platform to generate VLPs, the mechanistic understanding about polyethylenimine (PEI)-mediated plasmid DNA delivery is still unclear. The branched PEI-25K was demonstrated to be efficient for transient transfection [73]. However, cytotoxicity might limit its applications in large-scale production. In our laboratory, 293F cell viability decreased over time and reached less than 50% at 96 h post-transfection. A few studies suggested that the use of PEI-7K might reduce the cytotoxicity, compared with PEI-25K [75]. The transfection efficiency of plasmid DNA containing L1:P18I10 DNA coding sequence in 293F cells could be highly determined by DNA/PEI complexes in a DNA/PEI ratio-dependent manner [74,76]. Larger DNA/PEI complexes (>1 µm) through partially aggregation would be favorable to endocytosis of plasmid DNA, and contribute to high transfection efficiency [74,77].

Regarding to VLP purification methods from former studies, the recovery of HPV16 L1 VLPs using 40% or 45% sucrose cushion (SC) is 27% and 18.1%, respectively, and the purity is ranged between 2.2 and 5.4% [29]. Our ultracentrifugal processes using 70% SC resulted in 15% recovery and 6% purity of L1:P18I10 VLPs. These patterns suggested that the higher percentage of SC could increase purity but reduce recovery. We found that L1:P18I10 VLPs formed a distinctive band at the interface layer between 20% and 70% sucrose. This pattern was well in line with previous studies observing that HPV16 L1 VLPs form a visible band at a concentration of 30–40% in a continuous sucrose gradient [41]. As the HPV16 L1 VLPs are hollow interior and might have DNA-capsid affinity [78,79], SC-purified L1:P18I10 VLPs might encapsulate DNA and lead to irregular or heterogeneous forms [80]. Although heterogeneous L1:P18I10 VLPs could be further separated by CsCl density gradient, CsCl-purified L1:P18I10 VLPs were distributed in the whole gradient and heterogeneous in size due to DNA encapsulation [80] or broken particles [81]. In general, we observed that the high purity (>99%) of homogenous L1:P18I10 VLPs would be presented as a visible band in the CsCl gradient. Since HPV16 VLPs constructed by L1-based capsid proteins might be less efficient self-assembly than L1:L2 VLPs [22,25], different properties of chimeric L1:P18I10 VLPs compared to native HPV16 virions could be more fragile during purification. Therefore, ultracentrifugal approaches might provide a relatively mild purification condition and in favor of in vitro VLP stability and formation during purification process. It should be noted that the use of ultracentrifugation imposes a limit on the volume of cell lysates, which makes this protocol unsuitable for significant scale-up.

The recovery of L1:P18I10 VLPs during downstream purification is critical, because it affects overall costs in bioprocessing [82]. To obtain high purity of HPV16 L1 VLPs, multiple chromatographic steps might be required [29]. However, repeated procedures might affect VLP conformation and reduce the final recovery of VLPs. HPV16 L1 proteins generated from yeast were successfully purified using size exclusion (SEC), heparin-affinity (H-AC) or ion exchange (IEX) chromatography [27,28,29]. Recently, our research group demonstrated that chimeric HPV:HIV (L1:P18I10) proteins could successfully produced in *Pichia pastoris* yeast. After chromatographic and ultracentrifugal purification process, the L1:P18I10 VLPs were recovered with 96% purity and 9.23% overall recovery [57]. According to Merck’s data, recovery of HPV11 L1 VLPs after CEC is between 25 and 45% [83]. Another study reported that 63% recovery of HPV16 L1 VLPs by using CEC is achievable [28]. Since we fractionated L1:P18I10 VLP samples by one-step gradient elution, our data revealed a relatively higher recovery (~65%) but lower purity of L1:P18I10 VLPs after CEC. Because CEC matrices rely on diffusion-limited mass transfer, large molecular complexes such as L1:P18I10 VLPs might significantly reduce the column‘s overall dynamic binding capacity [42]. We showed that the recovery of L1:P18I10 VLPs after SEC is around 89% (from 65% of CEC step to 58% of SEC step) and is in concordance with the result presented in the GE Capto Core 700 application note [52]. Heparin was reported to interact with the intact conformation and properly folded HPV16 L1 VLPs because of its structurally similarity to heparan sulfate, which is related to HPV infection pathway [27,83]. Our data suggested that L1:P18I10 VLPs could also bind heparin. Because performance of heparin affinity chromatography and CEC to separate HPV16 L1 VLP are quite similar [28], removal of contaminants from L1:P18I10 VLPS by an additional heparin polishing step seems inefficient. All in all, our three-step chromatographic protocols verified that it is feasible for mammalian cell-derived L1:P18I10 VLPs purification with relatively higher recovery of 57%.

In our present study, we established optimum production and purification methods to engineer chimeric HPV:HIV (L1:P18I10) VLPs. Although the BEVS/IC system was widely used in licensed HPV prophylactic vaccine (Cervarix, Merck & Co., Rahway, NJ, USA) manufacturing, low expression level of L1 capsid proteins remained challenging for the production of certain HPV types [47] or as yet untargeted HPV:HIV VLPs. Here, we demonstrated that the mammalian cell (293F)-based expression system could be comparable method with BEVS/IC system regarding L1:P18I10 protein expression. Moreover, we proposed a simple one-step gradient elution protocol which is suited to the laboratory unequipped with advanced fast protein liquid chromatography (FPLC) system and can be used as a starting point to optimize chromatographic purification conditions for chimeric L1:P18I10 VLPs. The small-scale and three-step chromatographic purification method gave a significantly higher recovery of L1:P18I10 VLPs in comparison with conventional ultracentrifugal purification methods. There are still several bioprocessing challenges of chromatography, such as the maintenance of morphological properties of L1:P18I10 VLPs. Therefore, ultracentrifugal approaches are still irreplaceable to be used as standard VLP purification methods. In the future, it is expected that the 293F expression system combining with chromatography could be a scalable approach to engineer chimeric L1:P18I10 VLPs or other enveloped VLPs for industrial VLP-based vaccine manufacturing. This work contributes towards developing an alternative platform for production and purification of a bivalent VLP-based vaccine against HPV and HIV-1, which is urgently needed in developing and industrialized nations.

## 4. Materials and Methods

### 4.1. Cell Lines and Cell Culture

The insect Spodoptera frugiperda 9 (Sf9) cells (Gibco, Waltham, MA, USA) were grown in Grace’s insect medium (Gicbo), supplemented with 10% fetal bovine serum (FBS) (Sigma) and 100 U/mL of penicillin-streptomycin (Gibco), and incubated in a 27 °C incubator without a humidified atmosphere and CO_2_. The 293F cells (Gibco), derived from human embryonic kidney (HEK) 293 cells, were cultured in FreeStyle 293 expression medium (Gibco) supplemented with 5 mL/L of penicillin-streptomycin (Gibco) and incubated in a 37 °C incubator containing a humidified atmosphere of 5% CO_2_ on an orbital shaker platform rotating at 125 rpm.

### 4.2. Production of L1:P18I10 Proteins by Using BEVS/IC System

The HIV-1 P18I10 CTL peptide (RGPGRAFVTI) was inserted into the DE loop of HPV16 L1 capsid protein. HPV16 L1 DE loop sequence encoding 130–136 amino acids was replaced with P18I10I10 peptide. The recombinant baculoviruses were produced according to the manufacturer’s instructions of Bac-to-Bac BEVS/IC system with pFastBac kit (Invitrogen, Waltham, MA, USA). In brief, the chimeric L1:P18I10 DNA coding sequence was cloned into a baculovirus donor plasmid (pFastBac1) and transformed into competent *E. coli*. DH10Bac contains a parent bacmid with a lacZ-mini-attTn7 fusion. When the transposition was successful, the Sf9 cells were transfected with isolated DNA to produce first generation of recombinant baculovirus. The viral titer of amplified recombinant baculovirus was determined by plaque assay. A density of 1 × 10^6^/mL of Sf9 cells (~90% confluent) was seeded in 75 cm^2^ flask (Corning, New York, NY, USA) with 10 mL Grace’s insect/TNM-FH medium and infected with recombinant baculovirus at a multiplicity of infection (MOI) of 1 to generate the chimeric L1:P18I10 proteins. After 96 h post-infection, the Sf9 cell density can be about 1.2 to 1.5 × 10^6^ cells/mL with at least 20% viability.

### 4.3. Production of L1:P18I10 Proteins by Using 293F Expression System

The HIV-1 P18I10 CTL peptide (RGPGRAFVTI) was inserted into the DE loop of HPV16 L1 capsid protein. HPV16 L1 DE loop sequence encoding 130–136 amino acids was replaced with P18I10I10 peptide. The L1:P18I10 DNA coding sequence was modified with Kozak sequence, optimized with human codon, flanked by the restriction enzyme sites of HindIII and XbaI and cloned into pcDNA3.1(+) vector by using GeneArt gene synthesis services (Thermo Fisher, Waltham, MA, USA). The recombinant plasmid DNA (pDNA) was transformed into DH5α competent cells (Invitrogen) for amplification and extracted by using plasmid Maxi kits (QIAGEN, Hilden, Germany). The 293F cells were cultured with 30 mL FreeStyle 293 expression medium in a 125 mL Erlenmeyer flask (Corning) to a density of 1.0 × 10^6^/mL and transiently transfected with L1:P18 I10 pDNAs using the branched polyethylenimine with a MW of 25 kDa (PEI-25K) (Polysciences, Warrington, PA, USA) at an optimized ratio of DNA to PEI 1:3 (*w*/*w*) and DNA to culture medium 1:1 (*w*/*v*) according to manufacturer’s instructions [73]. The 293F cells were harvested at 96 h post-transfection. A total of 293F cells can reach a confluent density of 3.6 × 10^6^ cells/mL with around 50% viability.

### 4.4. Immunofluorescence Staining

We collected 100 μL culture medium (= total 1.2 × 10^5^ Sf9 cells) from a total of 1.2 × 10^7^ infected Sf9 cells in a 75 cm^2^ flask (10 mL culture medium/flask). Additionally, we collected 100 μL culture medium (= total 3.7 × 10^5^ 293F cells) from ~1.1 × 10^8^ transfected 293F cells in a 125 mL Erlenmeyer flask (30 mL culture medium/flask). The cells were permeabilized on the glass slide with 100% cold acetone. Subsequently, the fixed cells were probed with anti-HPV16 L1 antibody CAMVIR-1 (Abcam, Cambridge, UK) and captured with anti-mouse IgG-FITC (Sigma, St. Louis, MO, USA). Immune-stained cell monolayers were thoroughly washed with PBS and covered with mounting medium with DAPI (Abcam). The immunofluorescence images were inspected under an inverted microscope at 40× magnification. Transfection efficiency was determined by the ratio of FITC (green)-positive cells to DAPI (blue)-stained cells.

### 4.5. Cell Lysis and Clarification

A total of 1.0 × 10^8^ (~ 9.6 × 10^7^) infected Sf9 cells in eight 75 cm^2^ flasks (10 mL culture medium/flask) or 1.0 × 10^8^ (~1.1 × 10^8^) transfected 293F cells in a 125 mL Erlenmeyer flask (30 mL culture medium/flask) were collected by centrifugation at 1500 rpm for 5 min and washed twice with PBS. The pellet was re-suspended with cell lysis buffer formulated with 20 mM Tris (pH = 7.1), 20 mM NaCl, 1% Triton X-100, protease inhibitor (1:100) (Millipore, St. Louis, MO, USA), and Benzonase (25 U/mL) (Millipore) and 2 mM MgCl_2_ and was then incubated at 4 °C for 24 h for complete lysis and DNA degradation. The crude cell lysates were centrifuged at 13,000 rpm for 10 min at 4 °C, and, subsequently, clarified by the 0.45 µm syringe filter (Millipore) to remove cell debris and clumps.

### 4.6. Sucrose Cushion (SC)

The 20% and 70% sucrose cushions (SC) (*w*/*v*) were prepared in PBS (pH = 7.4, 137 mM NaCl). The clarified Sf9 cell lysate adjusted in PBS was carefully layered on the top of two-step (20% and 70%) sucrose cushion in a thin wall ultracentrifugation tube (Beckman, Brea, CA, USA). After ultracentrifugation at 40,000 rpm (274,000× *g*) for 4 h at 4 °C, the tube was placed on ice to avoid the interface layer being re-suspended. The practically purified and concentrated L1:P18I10 VLP sample was located by UV light and collected through a puncture using a 1 mL sterile syringe.

### 4.7. Cesium Chloride (CsCl) Density Gradient

The SC-purified L1:P18I10 VLP sample was mixed with 40% CsCl solution in PBS and scattered with the sonicator (Branson, New Haven, CT, USA). The L1:P18I10 VLP sample was ultracentrifuged at 40,000 rpm (274,000× *g*) for 16–24 h at 4 °C. After ultracentrifugation, the tube was placed on ice to avoid the layer being re-suspended. The CsCl gradient was fractionated from the top of the tube (400 μL per fraction). The signal of L1:P18I10 VLPs in each fraction was detected by dot blot, using anti-HPV16 L1 mAb CAMVIR-1.

### 4.8. Cation Exchange Chromatography (CEC)

The HiTrap Capto SP ImpRes column (1 mL, GE) was washed with 5 mL of ddH_2_O and equilibrated with 10 mL of the starting buffer (20 mM Tris-HCl, 100 mM NaCl, pH 7.1). Since we used HiLoad Pump P-50 (GE), pH and conductivity of flowthrough (FT), eluates or eluents were determined by pH papers (Sigma) and the EC/salinity meter. The clarified 293F cell lysate were adjusted to a volume of 5 mL with the starting buffer and loaded on to the column at a flow rate of 1 mL/min. After washing with 10 mL of the starting buffer, the CEC-captured L1:P18I10 VLPs were one-step eluted with 5 mL of elution buffers (20 mM Tris-HCl, 1 M NaCl, pH 7.1) at a flow rate of 1 mL/min. The column was regenerated by washing with 5 mL of 2 M NaCl in 20 mM Tris-HCl buffer at pH 7.1 to remove remaining ionically bound proteins.

### 4.9. Size Exclusion Chromatography (SEC)

Before using HiTrap Capto Core 700 column (1 mL, GE), cleaning-in-place (CIP) was performed to remove the bound impurities. The column was washed with 5 mL of ddH_2_O and equilibrated with 10 mL of the running buffer (20 mM Tris-HCl, 220 mM NaCl, pH 7.1). The CEC-purified L1:P18I10 VLP sample was adjusted to a volume of around 20 mL with the running buffer and loaded on to the column at a flow rate of 0.6 mL/min. The first and second fractions (10 mL per fraction) containing the SEC-purified L1:P18I10 VLPs were collected through washing with the running buffer. Finally, a CIP procedure was performed again to clean the column.

### 4.10. Heparin-Affinity Chromatography (H-AC)

The HiTrap Heparin HP column (1 mL, GE) was washed with 5 mL of ddH_2_O and equilibrated with 10 mL of the binding buffer (20 mM Tris-HCl, 220 mM NaCl, pH 7.1). The SEC-purified L1:P18I10 VLP sample (10 mL) was loaded on to the column at a flow rate of 0.5 mL/min. After washing with 10 mL of the binding buffer, the heparin-bound L1:P18I10 VLPs were one-step eluted with 5 mL of elution buffers (20 mM Tris-HCl, 1 M NaCl, pH 7.1) at a flow rate of 1 mL/min. The column was regenerated by washing with 5 mL of 2 M NaCl in 20 mM Tris-HCl buffer at pH 7.1 to remove remaining impurities. The eluates were subsequently diafiltrated 10-folds with Tris-HCl (pH = 7.4, 137 mM NaCl) by using 100 kDa Ultra-4 centrifugal filter devices (Amicon, Miami, FL, USA).

### 4.11. Non-Reducing SDS-PAGE

The L1:P18I10 VLPs purified by ultracentrifugal and chromatographic methods were mixed with 2× Laemmli sample buffer (BIO-RAD, Hercules, CA, USA) in the presence or absence of 20 mM dithiothreitol (DTT) and reacted at room temperature (RT) for 15 min. Samples were separated by 8–16% TGX stain-free protein gels (BIO-RAD). Then, the gels were transfer to PVDF membranes. The membranes were probed with the anti-HPV16 L1 CAMVIR-1 mAb at a dilution of 1:4000. After that, the membranes were incubated with anti-mouse IgG Peroxidase Conjugate (Sigma-Aldrich, St. Louis, MO, USA) at a dilution of 1:4000. The signal was developed and visualized by chemoluminiscence using Western Blot ECL substrate kit (Bio-Rad). The blot images were acquired by using Odyssey Fc imaging system.

### 4.12. Molecular Mass Analysis

L1:P18I10 VLPs purified from both methods were treated or un-treated with 20 mM DTT for 15 min and were then filtered out through 1000 kDa (SARTORIUS, Göttingen, Germany) or 100 kDa (Amicon) molecular weight cutoff (MWCO) ultrafiltration devices. The retentates were reconstituted to the original volume and collected from the filter device sample reservoir, while the filtrates were collected at the bottom of the centrifuge tube. The L1 signal was measured by using dot blot probed with anti-HPV16 L1 mAb and detected by anti-mouse IgG-peroxidase conjugate (Sigma-Aldrich). Images were acquired using Odyssey Fc Imaging System at a chemiluminescence channel.

### 4.13. Negative Staining and Transmission Electron Microscope

After charging the carbon-coated copper grids (Sigma-Aldrich) under ultraviolet light for 5 min, purified L1:P18I10 VLPs were absorbed on grids for 1 min and rinsed 3 times by miliQ water. The L1:P18I10 VLPs purified by ultracentrifugation in PBS (pH = 7.4, 137 mM NaCl) were negative-stained with 2% phosphotungstic acid (PTA) at pH 7.0 (Sigma-Aldrich) for 1 min. The L1:P18I10 VLPs purified from chromatography in Tris-HCl (pH = 7.4, 137 mM NaCl) were negative-stained with 2% uranyl acetate at pH 4.5 (Sigma-Aldrich) for 1 min. Excess staining agents were removed by Whatman qualitative filter paper (Sigma-Aldrich). Grids were placed in a dehumidifier chamber at least 2 h before observation. Images were acquired using a transmission electron microscopy (Tecnai Spirit 120 kV, FEI Company, Hillsboro, OR, USA) at magnification SA270K (50 nm), SA59000 (200 nm) and SA529500 (400 nm), respectively.

### 4.14. Quantification of L1:P18I10 VLPs and Host Cellular Proteins

The band intensity of L1 from the Western blot was quantified by densitometric assay using Image Studio Lite version 5.x software. The purified L1:P18I10 VLPs were quantified by indirect ELISA. The band intensity of total host cellular proteins (HCPs) from Coomassie-stained SDS-PAGE was quantified by densitometric assay using Image Studio Lite 5.x software. The HCPs were also quantified by BCA protein assay kit (Thermo Fisher). 2 mg/mL of bovine serum blbumin Standard (Thermo Fisher) were used to construct a standard curve plotting concentration versus absorbance. The total protein from each purification step was extrapolated from this standard curve to determine the actual amount of HCPs by using a NanoDrop 2000 spectrophotometer.

### 4.15. Western Blotting Analysis

Equal amounts (200 ng) of HPV16 L1 protein (Abcam) and L1:P18I10 VLPs purified from both methods were mixed with 2× Laemmli sample buffer containing 5% 2-ME and boiled at 95 °C for 5 min. Samples were separated by 8–16% TGX Stain-free protein gels and were then transferred to a PVDF membrane (Millipore) using a Semi-Dry transfer device (Bio-Rad). The membrane was blocked with 5% skim milk in TBST. Then, the membranes were probed with the anti-HPV16 L1 CAMVIR-1 mAb at a dilution of 1:4000 and anti-HIV1-V3 loop mAb (NIBSC, EVA3013) at a dilution of 1:500, respectively. After that, the membranes were incubated with anti-mouse IgG Peroxidase Conjugate (Sigma-Aldrich) at a dilution of 1:4000. The signal was developed and visualized by chemoluminiscence using Western Blot ECL substrate kit (Bio-Rad). The blot images were acquired by using Odyssey Fc imaging system.

### 4.16. Immunization of Mice and Sample Collection

Chromatography-purified L1:P18I10 VLPs were emulsified with an equal volume of (225 μg per each 0.5 mL dose) aluminum hydroxyphosphate sulfate (Thermo Fisher), to ensure a similar formulation to the licensed Gardasil-9 HPV vaccine [84]. All mouse groups had equal gender distribution (male *n* = 4 and female *n* = 4 per group). In the group A, BALB/c mice were immunized intramuscularly (i.m.) with 10 μg of L1:P18I10 VLPs, respectively by following a homologous prime-boost regime. In the group B, positive control mice were offered Gardasil-9 prime followed by Gardasil-9 boost intramuscularly with 10 μg of HPV16 L1 VLPs. The prime-boost interval was 2 weeks. Mice were sacrificed on day 28. Blood samples were collected from the heart of mice. Sera were recovered by centrifugation and stored at −20 °C for ELISA assay. Murine spleens were removed and pressed individually through a cell strainer (Falcon) with a 5 mL syringe rubber plunger. Following the removal of red blood cells with ACK lysing buffer (Lonza), splenocytes were washed and resuspended in lymphocyte medium R10 (RPMI 1640 supplemented with 10% fetal calf serum (FCS), penicillin-streptomycin, 20 mM HEPES and 15 mM 2-ME) at a concentration of 2 × 10^7^ cells/mL.

### 4.17. Enzyme-Linked Immunosorbent Assay (ELISA)

To measure the VLP-induced antibodies in the sera of BALB/c mice, the microtiter plates were coated with 50 µL of 2 µg/mL HPV16 L1 VLPs and HIV-1 P18I10 peptide (NIBSC, ARP734), respectively, with 50 mM carbonate–bicarbonate buffer (pH = 9.6). The plates were incubated at 4 °C overnight. Plates were blocked with the blocking buffer (5% skim milk in TBST) at 37 °C for at least 2 h. At the same time, sera collected from group A and B immunized mice were two-fold serially diluted with 5% skim milk in TBST from a ratio of 1:50 to 1:800. After washing twice with TBST, the plates were added with the diluted sera and incubated at 37 °C for 2 h. After washing 3 times with TBST, the plates were added with recombinant protein G HRP conjugate at a dilution of 1:4000 in blocking buffer and incubated at 37 °C for 1 h. TMB was used to develop the ELISA signal and stopped with 50 µL of 2 M H_2_SO_4_. The OD of each well was measured at a wavelength of 450 nm by using ELx800 absorbance microplate reader.

### 4.18. Mouse IFN-γ Enzyme-Linked Immunosorbent Spot Assay (ELISpot)

The ELISpot assay was performed according to the manufacturer’s instructions (Mabtech, Nacka Strand, Sweden). The PVDF plates (MSISP4510, Millipore) pre-treated with 70% EtOH were coated with anti-mouse IFN-γ capture mAb (15 μg/mL) in PBS and incubated at 4 °C overnight. After removing excess antibody by washing 5 times with PBS, a total of 2.5 × 10^5^ fresh splenocytes were added to each well. Subsequently, the cells from group A and B were stimulated with 2 μg/mL of HPV16 L1 VLPs and HIV-1 P18I10 peptides, respectively, and the plates were incubated at 37 °C with 5% CO_2_ for 24 h. After emptying the cells by washing 5 times with PBS, the plates were added with biotinylated anti-IFN-γ detection mAb diluted to a concentration of 1 μg/mL in PBS containing 0.5% FCS and incubated for 2 h at RT. After washing 5 times with PBS, the plates were added with diluted Streptavidin-ALP (1:1000) in PBS-0.5% FCS and incubated for 1 h at RT. After the final wash, the alkaline phosphatase conjugate substrate (BIO-RAD) was added to the plate until distinct spots emerge. Color development was stopped by washing extensively in tap water, and the count spots were inspected using an ELISpot reader (AID Autoimmun Diagnostika GmbH, Straßberg, Germany).

### 4.19. Statistical Analysis

Statistical analysis was performed using Prism 6 GraphPad software (San Diego, CA, USA). The line graph of ELISAs were analyzed by simple linear regression test to compare the slope of the two lines together and to confirm two data sets were significant different. ELISpot data were tested by unpaired *t* test to determine the statistical significance between two groups.

### 4.20. Mice and Ethics Statements

Six to eight-week-old BALB/c mice were purchased from Envigo (Indianapolis, IN, USA) and approved by local authorities (Generalitat de Catalunya, project number 11157) and the Universitat Autònoma de Barcelona Ethics Commitee. The animal welfare legislation strictly conformed to the Generalitat de Catalunya. All experimental works were approved by the local Research Ethics Committee (Procedure 43.19, Hospital de la Vall d’Hebron, Universitat Autònoma de Barcelona).

## Figures and Tables

**Table 1:** Purification profiles of L1:P18I10 VLPs.

Method	Purification Stages	Total HCP (μg) ^a^	HCP Removal (%)	Total L1 (μg) ^b^	Recovery (%)	Purity (%) ^d^
1 Traditional	BEVS/IC CCL	2637.4	-	109.4	100	4
	Ultracentrifugation					
	Step-1: SC	264.5	90	16.6	15	6
	Step-2: CsCl	12.0	99	11.9	11	99
2 New	293F cell expression CCL	1848.5	-	33.3	100	2
	Chromatography					
	Step-1: CEC	649.2	65	21.5	65	3
	Step-2: SEC	193.9	90	19.4 ^c^	58	10
	Step-3: H-AC	24.7	98	18.8 ^c^	56	76

^a^: Determined by BCA assay; ^b^: Determined by densitometry of Western blot; ^c^: Determined by ELISA. ^d^: Determined by a ratio of total L1 to total HCP. Abbreviations: CCL: clarified cell lysate; SC: sucrose cushion; CsCl: CsCl density gradient; CEC: cation exchange chromatography; SEC: size exclusion chromatography; H-AC: heparin-affinity chromatography.

## Data Availability

All data are contained within the article.

## References

[1] (2019). UNAIDS Global HIV & AIDS Statistics—2018 Fact Sheet. http://www.unaids.org/en/resources/fact-sheet.

[2] Wang Q., Zhang L. (2020). Broadly neutralizing antibodies and vaccine design against HIV-1 infection. Front. Med..

[3] Streeck H., D’souza M.P., Littman D.R., Crotty S. (2013). Harnessing CD4 + T cell responses in HIV vaccine development. Nat. Med..

[4] Collins D.R., Gaiha G.D., Walker B.D. (2020). CD8+ T cells in HIV control, cure and prevention. Nat. Rev. Immunol..

[5] Rerks-Ngarm S., Pitisuttithum P., Nitayaphan S., Kaewkungwal J., Chiu J., Paris R., Premsri N., Namwat C., De Souza M., Adams E. (2009). Vaccination with ALVAC and AIDSVAX to prevent HIV-1 infection in Thailand. N. Engl. J. Med..

[6] Ng’uni T., Chasara C., Ndhlovu Z.M. (2020). Major scientific hurdles in HIV vaccine development: Historical perspective and future directions. Front. Immunol..

[7] Robinson H.L. (2018). HIV/AIDS vaccines: 2018. Clin. Pharmacol. Ther..

[8] Ahmed H.G., Bensumaidea S.H., Alshammari F.D., Alenazi F.S.H., Almutlaq B.A., Alturkstani M.Z., Aladani I.A. (2017). Prevalence of human papillomavirus subtypes 16 and 18 among Yemeni patients with cervical cancer. Asian Pacific J. Cancer Prev..

[9] Olcese V.A., Chen Y., Schlegel R., Yuan H. (2004). Characterization of HPV16 L1 loop domains in the formation of a type-specific, conformational epitope. BMC Microbiol..

[10] Dupuy C., Buzoni-Gate D., Touze A., Le Cann P., Bout D., Coursaget P. (1997). Cell mediated immunity induced in mice by HPV 16 L1 virus-like particles. Microb. Pathog..

[11] Schiller J., Lowy D. (2018). Explanations for the high potency of HPV prophylactic vaccines. Vaccine.

[12] Markowitz L.E., Schiller J.T. (2021). Human Papillomavirus Vaccines. J. Infect. Dis..

[13] Chen C.W., Saubi N., Joseph-Munné J. (2020). Design concepts of virus-like particle-based HIV-1 vaccines. Front. Immunol..

[14] Eto Y., Saubi N., Ferrer P., Joseph J. (2019). Designing chimeric virus-like particle-based vaccines for human papillomavirus and HIV: Lessons learned. AIDS Rev..

[15] Liu W.J., Liu X.S., Zhao K.N., Leggatt G.R., Frazer I.H. (2000). Papillomavirus virus-like particles for the delivery of multiple cytotoxic T cell epitopes. Virology.

[16] Liu X.S., Abdul-Jabbar I., Qi Y.M., Frazer I.H., Zhou J. (1998). Mucosal immunisation with papillomavirus virus-like particles elicits systemic and mucosal immunity in mice. Virology.

[17] Peng S., Frazer I.H., Fernando G.J., Zhou J. (1998). Papillomavirus virus-like particles can deliver defined CTL epitopes to the MHC class I pathway. Virology.

[18] Xiao S.L., Wen J.L., Kong N.Z., Yue H.L., Leggatt G., Frazer I.H. (2002). Route of administration of chimeric BPV1 VLP determines the character of the induced immune responses. Immunol. Cell Biol..

[19] Zhai Y., Zhong Z., Zariffard M., Spear G.T., Qiao L. (2013). Bovine papillomavirus-like particles presenting conserved epitopes from membrane-proximal external region of HIV-1 gp41 induced mucosal and systemic antibodies. Vaccine.

[20] Zhang H., Huang Y., Fayad R., Spear G.T., Qiao L. (2004). Induction of mucosal and systemic neutralizing antibodies against human immunodeficiency virus type 1 (HIV-1) by oral immunization with bovine papillomavirus-HIV-1 gp41 chimeric virus-like particles. J. Virol..

[21] Chen C.-W., Saubi N., Kilpeläinen A., Joseph-Munné J. (2022). Chimeric Human Papillomavirus-16 Virus-like Particles Presenting P18I10 and T20 Peptides from HIV-1 Envelope Induce HPV16 and HIV-1-Specific Humoral and T Cell-Mediated Immunity in BALB/c Mice. Vaccines.

[22] Kirnbauer R., Taub J., Greenstone H., Roden R., Dürst M., Gissmann L., Lowy D.R., Schiller J.T. (1993). Efficient Self-Assembly of Human Papillomavirus Type 16 LI and L1-L2 into Virus-like Particles. J. Virol..

[23] Zhao Q., Potter C.S., Carragher B., Lander G., Sworen J., Towne V., Abraham D., Duncan P., Washabaugh M.W., Sitrin R.D. (2014). Characterization of virus-like particles in GARDASIL^®^ by cryo transmission electron microscopy. Hum. Vaccines Immunother..

[24] Le Cann P., Coursaget P., Iochmann S., Touze A. (1994). Self-assembly of human papillomavirus type 16 capsids by expression of the L1 protein in insect cells. FEMS Microbiol. Lett..

[25] Sasagawa T., Pushko P., Steers G., Gschmeissner S.E., Nasser Hajibagheri M.A., Finch J., Crawford L., Tommasino M. (1995). Synthesis and assembly of virus-like particles of human papillomaviruses type 6and Type 16 in fission yeast *Schizosaccharomyces pombe*. Virology.

[26] Kim S.N., Jeong H.S., Park S.N., Kim H.J. (2007). Purification and immunogenicity study of human papillomavirus type 16 L1 protein in *Saccharomyces cerevisiae*. J. Virol. Methods.

[27] Kim H.J., Kim S.Y., Lim S.J., Kim J.Y., Lee S.J., Kim H.J. (2010). One-step chromatographic purification of human papillomavirus type 16 L1 protein from *Saccharomyces cerevisiae*. Protein Expr. Purif..

[28] Park M.A., Kim H.J., Kim H.J. (2008). Optimum conditions for production and purification of human papillomavirus type 16 L1 protein from *Saccharomyces cerevisiae*. Protein Expr. Purif..

[29] Zhang W., Carmichael J., Ferguson J., Inglis S., Ashrafian H., Stanley M. (1998). Expression of human papillomavirus type 16 L1 protein in *Escherichia coli*: Denaturation, renaturation, and self-assembly of virus-like particles in vitro. Virology.

[30] Schädlich L., Senger T., Kirschning C.J., Müller M., Gissmann L. (2009). Refining HPV 16 L1 purification from *E. coli*: Reducing endotoxin contaminations and their impact on immunogenicity. Vaccine.

[31] Chen X.S., Casini G., Harrison S.C., Garcea R.L. (2001). Papillomavirus capsid protein expression in *Escherichia coli*: Purification and assembly of HPV11 and HPV16 L1. J. Mol. Biol..

[32] Aires K.A., Cianciarullo A.M., Carneiro S.M., Villa L.L., Boccardo E., Pérez-Martinez C., Perez-Arellano I., Oliveira M.L.S., Ho P.L. (2006). Production of human papillomavirus type 16 L1 virus-like particles by recombinant *Lactobacillus casei* cells. Appl. Environ. Microbiol..

[33] Biemelt S., Sonnewald U., Galmbacher P., Willmitzer L., Müller M. (2003). Production of Human Papillomavirus Type 16 Virus-Like Particles in Transgenic Plants. J. Virol..

[34] Zahin M., Joh J., Khanal S., Husk A., Mason H., Warzecha H., Ghim S.J., Miller D.M., Matoba N., Jenson A.B. (2016). Scalable production of HPV16 L1 protein and VLPs from tobacco leaves. PLoS ONE.

[35] Fuenmayor J., Gòdia F., Cervera L. (2017). Production of virus-like particles for vaccines. N. Biotechnol..

[36] Vicente T., Roldão A., Peixoto C., Carrondo M.J.T., Alves P.M. (2011). Large-scale production and purification of VLP-based vaccines. J. Invertebr. Pathol..

[37] Monie A., Hung C.F., Roden R., Wu T.C. (2008). Cervarix^TM^:A vaccine for the prevention of HPV 16, 18-associated cervical cancer. Biol. Targets Ther..

[38] Monograph P. (2010). CERVARIX–Product monograph. Toxicology.

[39] Nooraei S., Bahrulolum H., Hoseini Z.S., Katalani C., Hajizade A., Easton A.J., Ahmadian G. (2021). Virus-like particles: Preparation, immunogenicity and their roles as nanovaccines and drug nanocarriers. J. Nanobiotechnology.

[40] Millipore Sigma (2016). Generic Process of Virus-like Particle (VLP) Based Vaccine Manufacturing.

[41] Vlps A., Middelberg A.P.J., Lua L.H.L. (2013). Virus-like particle bioprocessing: Challenges and opportunities. Pharm. Bioprocess..

[42] Achour A., Lemhammedi S., Picard O., M’bika J.P., Zagury J.F., Moukrim Z., Willer A., Beix F., Burny A., Zagury D. (1994). Cytotoxic T Lymphocytes Specific for HIV-1 gp160 Antigen and Synthetic P18IIIB Peptide in an HLA-A11-Immunized Individual. AIDS Res. Hum. Retrovir..

[43] Nakagawa Y., Kikuchi H., Takahashi H. (2007). Molecular analysis of TCR and peptide/MHC interaction using P18-I10-derived peptides with a single D-amino acid substitution. Biophys. J..

[44] Chen X.S., Garcea R.L., Goldberg I., Casini G., Harrison S.C. (2000). Structure of Small Virus-like Particles Assembled from the L1 Protein of Human Papillomavirus 16. Mol. Cell.

[45] McLean C.S., Churcher M.J., Meinke J., Smith G.L., Higgins G., Stanley M., Minson A.C. (1990). Production and characterisation of a monoclonal antibody to human papillomavirus type 16 using recombinant vaccinia virus. J. Clin. Pathol..

[46] Senger T., Schädlich L., Gissmann L., Müller M. (2009). Enhanced papillomavirus-like particle production in insect cells. Virology.

[47] Peyret H. (2015). A protocol for the gentle purification of virus-like particles produced in plants. J. Virol. Methods.

[48] Yazdani R., Shams-Bakhsh M., Hassani-Mehraban A., Arab S.S., Thelen N., Thiry M., Crommen J., Fillet M., Jacobs N., Brans A. (2019). Production and characterization of virus-like particles of grapevine fanleaf virus presenting L2 epitope of human papillomavirus minor capsid protein. BMC Biotechnol..

[49] Park J.Y., Pyo H.M., Yoon S.W., Baek S.Y., Park S.N., Kim C.J., Poo H. (2002). Production and prophylactic efficacy study of human papillomavirus-like particle expressing HPV16 L1 capsid protein. J. Microbiol..

[50] Volpers C., Schirmacher P., Streeck R.E., Sapp M. (1994). Assembly of the Major and the Minor Capsid Protein of Human Papillomavirus Type 33 into Virus-like Particles and Tubular Structures in Insect Cells. Virology.

[51] (2014). The use of Capto^TM^ Core 700 and Capto Q ImpRes in the purification of human papilloma virus like particles. J. Asia’s Pharm. Biopharm. Ind..

[52] Mukherjee S., Thorsteinsson M.V., Johnston L.B., DePhillips P.A., Zlotnick A. (2008). A Quantitative Description of In Vitro Assembly of Human Papillomavirus 16 Virus-Like Particles. J. Mol. Biol..

[53] McCarthy M.P., White W.I., Palmer-Hill F., Koenig S., Suzich J.A. (1998). Quantitative Disassembly and Reassembly of Human Papillomavirus Type 11 Viruslike Particles In Vitro. J. Virol..

[54] Mistry N., Wibom C., Evander M. (2008). Cutaneous and mucosal human papillomaviruses differ in net surface charge, potential impact on tropism. Virol. J..

[55] Shank-Retzlaff M.L., Zhao Q., Anderson C., Hamm M., High K., Nguyen M., Wang F., Wang N., Wang B., Wang Y. (2006). Evaluation of the thermal stability of Gardasil^®^. Hum. Vaccin..

[56] Eto Y., Saubi N., Ferrer P., Joseph-Munné J. (2021). Expression of chimeric HPV-HIV protein L1P18 in *pichia pastoris*; purification and characterization of the virus-like particles. Pharmaceutics.

[57] Shi L., Sanyal G., Ni A., Luo Z., Doshna S., Wang B., Graham T.L., Wang N., Volkin D.B. (2005). Stabilization of human papillomavirus virus-like particles by non-ionic surfactants. J. Pharm. Sci..

[58] Carter J.J., Wipf G.C., Benki S.F., Christensen N.D., Galloway D.A., Al C.E.T., Irol J.V. (2003). Identification of a Human Papillomavirus Type 16-Specific Epitope on the C-Terminal Arm of the Major Capsid Protein L1. J. Virol..

[59] Von Brunn A., Brand M., Reichhuber C., Morys-wortmann C., Deinhardt F., Schdelt F. (1993). Principal neutralizing domain of HIV-I is highly immunogenic when expressed on the surface of hepatitis B core particles. Vaccine.

[60] Shank-Retzlaff M., Wang F., Morley T., Anderson C., Hamm M., Brown M., Rowland K., Pancari G., Zorman J., Lowe R. (2005). Correlation between mouse potency and in vitro relative potency for human papillomavirus Type 16 virus-like particles and Gardasil vaccine samples. Hum. Vaccin..

[61] Yang O.O., Kalams S.A., Trocha A., Cao H., Luster A., Johnson R.P., Walker B.D. (1997). Suppression of human immunodeficiency virus type 1 replication by CD8+ cells: Evidence for HLA class I-restricted triggering of cytolytic and noncytolytic mechanisms. J. Virol..

[62] Goulder P.J.R., Watkins D.I. (2008). Impact of MHC class I diversity on immune control of immunodeficiency virus replication. Nat. Rev. Immunol..

[63] Joseph J., Saubi N., Im E.J., Fernández-Lloris R., Gil O., Cardona P.J., Gatell J.M., Hanke T. (2011). Newborn mice vaccination with BCG.HIVA222 + MVA.HIVA enhances HIV-1-specific immune responses: Influence of age and immunization routes. Clin. Dev. Immunol..

[64] Saubi N., Gea-Mallorquí E., Ferrer P., Hurtado C., Sánchez-Úbeda S., Eto Y., Gatell J.M., Hanke T., Joseph J. (2014). Engineering new mycobacterial vaccine design for HIV-TB pediatric vaccine vectored by lysine auxotroph of BCG. Mol. Ther. Methods Clin. Dev..

[65] Mahant A., Saubi N., Eto Y., Guitart N., Gatell J.M., Hanke T., Joseph J. (2017). Preclinical development of BCG.HIVA2auxo.int, harboring an integrative expression vector, for a HIV-TB Pediatric vaccine. Enhancement of stability and specific HIV-1 T-cell immunity. Hum. Vaccines Immunother..

[66] Hanke T., McMichael A.J. (2000). Design and construction of an experimental HIV-1 vaccine for a year-2000 clinical trial in Kenya. Nat. Med..

[67] Clendinen C., Zhang Y., Warburton R.N., Light D.W. (2016). Manufacturing costs of HPV vaccines for developing countries. Vaccine.

[68] Deng F. (2018). Advances and challenges in enveloped virus-like particle (VLP)-based vaccines. J. Immunol. Sci..

[69] Hervas-Stubbs S., Rueda P., Lopez L., Leclerc C. (2007). Insect Baculoviruses Strongly Potentiate Adaptive Immune Responses by Inducing Type I IFN. J. Immunol..

[70] Rueda P., Fominaya J., Langeveld J.P.M., Bruschke C., Vela C., Casal J.I. (2000). Effect of different baculovirus inactivation procedures on the integrity and immunogenicity of porcine parvovirus-like particles. Vaccine.

[71] Longo P.A., Kavran J.M., Kim M.-S., Leahy D.J. (2013). Transient Mammalian Cell Transfection with Polyethylenimine (PEI). Methods Enzymol.

[72] Durocher Y., Perret S., Kamen A. (2002). High-level and high-throughput recombinant protein production by transient transfection of suspension-growing human 293-EBNA1 cells. Nucleic Acids Res..

[73] Fang X.T., Sehlin D., Lannfelt L., Syvänen S., Hultqvist G. (2017). Efficient and inexpensive transient expression of multispecific multivalent antibodies in Expi293 cells. Biol. Proced. Online.

[74] Deng R., Yue Y., Jin F., Chen Y., Kung H.F., Lin M.C.M., Wu C. (2009). Revisit the complexation of PEI and DNA—How to make low cytotoxic and highly efficient PEI gene transfection non-viral vectors with a controllable chain length and structure?. J. Control. Release.

[75] Oh Y.K., Suh D., Kim J.M., Choi H.G., Shin K., Ko J.J. (2002). Polyethylenimine-mediated cellular uptake, nucleus trafficking and expression of cytokine plasmid DNA. Gene Ther..

[76] Han X., Fang Q., Yao F., Wang X., Wang J., Yang S., Shen B.Q. (2009). The heterogeneous nature of polyethylenimine-DNA complex formation affects transient gene expression. Cytotechnology.

[77] Zhou J., Doorbar J., Sun X.Y., Crawford L.V., McLean C.S., Frazer I.H. (1991). Identification of the nuclear localization signal of human papillomavirus type 16 L1 protein. Virology.

[78] Day P.M., Weisberg A.S., Thompson C.D., Hughes M.M., Pang Y.Y., Lowy D.R., Schiller J.T. (2019). Human Papillomavirus 16 Capsids Mediate Nuclear Entry during Infection. J. Virol..

[79] Lipin D.I., Chuan Y.P., Lua L.H.L., Middelberg A.P.J. (2008). Encapsulation of DNA and non-viral protein changes the structure of murine polyomavirus virus-like particles. Arch. Virol..

[80] Huhti L., Blazevic V., Nurminen K., Koho T., Hytönen V.P., Vesikari T. (2010). A comparison of methods for purification and concentration of norovirus GII-4 capsid virus-like particles. Arch. Virol..

[81] Sarubbi E. (2005). Protein batches from downstream processing studies can be cost-effective tools for fast and specific protein quantification assays. Anal. Biochem..

[82] Cook J.C., Joyce J.G., George H.A., Schultz L.D., Hurni W.M., Jansen K.U., Hepler R.W., Ip C., Lowe R.S., Keller P.M. (1999). Purification of virus-like particles of recombinant human papillomavirus type 11 major capsid protein L1 from *Saccharomyces cerevisiae*. Protein Expr. Purif..

[83] Rommel O., Dillner J., Fligge C., Bergsdorf C., Wang X., Seiinka H.C., Sapp M. (2005). Heparan sulfate proteoglycans interact exclusively with conformationally intact HPV L1 assemblies: Basis for a virus-like particle ELISA. J. Med. Virol..

[84] Merck Canada Inc. (2015). Product Monograph Gardasil® Product Monograph Monopril * Product Monograph.

